# Novel G‐CSF conjugated anionic globular dendrimer: Preparation and biological activity assessment

**DOI:** 10.1002/prp2.826

**Published:** 2021-07-16

**Authors:** Seyed Shahaboddin Mousavi Motlagh, Mohammad Seyedhamzeh, Reza Ahangari Cohan, Mehdi Shafiee Ardestani, Behrouz Vaziri, Kayhan Azadmanesh, Sahar Saberi, Vahideh Masoumi

**Affiliations:** ^1^ Department of Nanobiotechnology New Technologies Research Group Pasteur Institute of Iran Tehran Iran; ^2^ Faculty of Pharmacy Tehran University of Medical Sciences Tehran Iran; ^3^ Biotechnology Research Center Pasteur Institute of Iran Tehran Iran; ^4^ Department of Molecular Virology Pasteur Institute of Iran Tehran Iran; ^5^ Department of Biotechnology, Food and Drug Control Laboratories National Food and Drug Organization Tehran Iran

**Keywords:** biodistribution, biological activity, conjugate, dendrimer, granulocyte colony‐stimulating factor, toxicity

## Abstract

The most crucial role of granulocyte colony‐stimulating factor (G‐CSF) in the body is to increase the strength of immune system. In recent years, research on the use of nanoparticles in pharmaceuticals has been considered, most of which have been for drug‐loading purposes. In this study, a novel G‐CSF conjugated dendrimer was synthesized and characterized using different techniques. In vitro cytotoxicity was assessed on A549 and L929 cells, while abnormal toxicity was studied in mice. In vitro and in vivo biological activities were assessed in NFS60 cells and rats, respectively. In addition, in vivo distribution, plasma half‐life, and histopathological effect were studied in rat. The characterization tests confirmed the successful conjugation. There was no difference between G‐CSF cytotoxicity before and after conjugation, and no difference with the control group. No mice showed abnormal toxicity. Although in vitro biological activity revealed both conjugated and free G‐CSF promote proliferation cells, biological activity decreased significantly after conjugation about one‐third of the unconjugated form. Nonetheless, in vivo biological activity of conjugated G‐CSF increased by more than 2.5‐fold relative to the unconjugated form, totally. Fortunately, no histopathologic adverse effect was observed in vital rat tissues. Also, in vivo distribution of the conjugate was similar to the native protein with an enhanced terminal half‐life. Our data revealed that G‐CSF conjugated dendrimer could be considered as a candidate to improve the in vivo biological activity of G‐CSF. Moreover, multivalent capability of the dendrimer may be used for other new potentials of G‐CSF in future perspectives.

## Graphical abstract

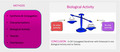

**Abbreviations**
AFM: atomic force microscopy
DCC: dicyclohexyl carbodiimide
DLS: dynamic light scattering
DMF: dimethylformamide
EGF: epidermal growth factor
EDC: N‐ethyl‐N′‐(3‐dimethylaminopropyl) carbodiimide
EDS‐Map: elemental analysis
FBS: fetal bovine serum
FESEM: field emission scanning electron microscope
FGF: Fibroblast growth factor
FTIR: fourier‐transform infrared spectroscopy
G‐CSF: granulocyte colony‐stimulating factor
G‐CSFR: granulocyte colony‐stimulating factor receptor
GMP: good manufacturing practice
GM‐CSF: granulocyte‐macrophage colony‐stimulating factor
IFN‐Beta: Interferon beta
IL‐1: Interleukin 1
IL1R: Interleukin‐1 receptor
IL‐3: Interleukin 3
IL‐5: Interleukin 5
IL‐7: Interleukin 7
LPS: Lipopolysaccharide
NHS: N‐hydroxysuccinimide
NIBSC: National Institute for Biological Standard and Control
PLGA: poly(lactic‐co‐glycolic acid)
SDS: sodium dodecyl sulfate
SDS‐PAGE: SDS‐polyacrylamide gel electrophoresis
SEC‐HPLC: size exclusion chromatography‐high performance liquid chromatography
SPECT: single‐photon emission computed tomography
TNF‐alpha: Tumor Necrosis Factor alpha
UV: ultraviolet
UV/Vis spectroscopy: ultraviolet‐visible spectroscopy
VEGF: Vascular endothelial growth factor
WHO: World Health Organization

## 1 INTRODUCTION

G‐CSF plays a significant role in the production of neutrophils in both the normal and pathological conditions.^1^ This glycoprotein is a member of colony‐stimulating factor family and the pharmacological effect is similar to GM‐CSF, IL‐3, and IL‐5. Fibroblasts, endothelial cells (related to bone marrow stroma) and cells involved in the immunity (monocytes and macrophages) are the main producing sources of this cytokine.^2^ However, many cells and tissues can produce G‐CSF if appropriately stimulated with VEGF, IL‐7, IL‐1, LPS, TNF‐alpha, and IFN‐Beta. The release of G‐CSF into the bloodstream affects the neutrophil population. It causes survival, proliferation, maturation, and movement of neutrophils from the bone marrow to the blood, and eventually to the body tissues. G‐CSF affects the function of neutrophils in situ and increases the motility of neutrophils in response to chemotaxis. It also acts as an inducer of anti‐inflammatory factors such as IL1R antagonists and TNF‐soluble receptors.^3^ Pharmacologically, G‐CSF exerts its effects by homodimerization of G‐CSF receptor. This membrane protein belongs to class I cytokine receptors and activates several downstream cytoplasmic tyrosine kinase pathways such as Jak/stat/Socs, Ras/Raf/Erk, and PI3kinase/Akt. The generated signals are terminated by negative regulators (Shp1 and Socs3) or internalization of the receptor and subsequent ubiquitination pathway.^4^ G‐CSF is used popularly to treat various neutropenia resulting from chemotherapy or severe congenital neutropenia. In addition, it has been used to call stem cells and blood precursors from the bone marrow to the peripheral blood to avoid bone marrow transplantation.^5^ An important medicinal effect of G‐CSF is also related to central nervous system disorders.^6^ G‐CSF administration is useful in acute neuronal degeneration and long‐term plasticity after cerebral ischemia.^7^ It exerts neuroprotective effects by reducing pro‐apoptotic proteins and increasing anti‐apoptotic proteins, which reduces mitochondrial stress. Other beneficial effects include: growth and migration of endothelial cells, balance in osteoclast and osteoblast activities, and reduced norepinephrine uptake.^8^ Eventually, it was found that Skeletal muscles increase their repair speed and even regeneration after injury, by increasing the G‐CSFR at their surface and receiving more G‐CSF.^9^

Different variants of G‐CSF have been developed for therapeutic purposes through amino acid addition, or substitution, chemical modifications with natural and synthetic polymers, and development of fusion forms. These researches were aimed to achieve more active molecules or longer lifespans.^9^, ^10^, ^11^, ^12^, ^13^

Nanotechnology is a promising field to modify biological macromolecules that often yielded positive results. Among nanoparticles, dendrimers have unique properties such as a globular structure and most importantly polyvalent properties that make them suitable agents in medicinal chemistry.^14^ For example, EGF and FGF glycoproteins are used for conjugation to the dendrimers.^15^, ^16^ In the case of EGF conjugate, it was found that cell growth could increase by 40% relative to the unconjugated form.^17^ Dendrimers often require surface modification to reduce toxicity and increase biocompatibility.^18^ For example, polyethylene glycol–citrate dendrimers have suitable biocompatible and biodegradable properties for medicinal purposes.^19^ Based on our knowledge, no G‐CSF modification with this dendrimer was reported so far. Therefore, in the current study, polyethylene glycol–citrate dendrimer was used to conjugate G‐CSF for investigation of effects on G‐CSF, especially its biological activity.

## 2 MATERIALS AND METHODS

Dicyclohexyl carbodiimide (DCC) (Cat # 802954), Dimethylformamide (DMF) (Cat # 103053), Poly (ethylene glycol) diacid (Cat # 843912), and anhydrous citric acid (Cat # 818707) were purchased from Merck. PD‐10 desalting columns containing Sephadex G‐25 resin (Cat # 170851) and Sephadex G15 (Cat # 170020) were purchased from GE Healthcare. N‐Hydroxysuccinimide (NHS) (Cat # 130672), RPMI 1640 (Cat # R8758), XTT (Cat # X4626), Hochest (Cat # B1155), and N‐Ethyl‐N′‐(3‐dimethylaminopropyl) carbodiimide (EDC) (Cat # E1769) were purchased from Sigma Aldrich. A549, L929, and NFS60 cell lines, rats, and mice were obtained from Iranian Genetic Resources Center and Pasteur Institute of Iran, respectively. G‐CSF was purchased from Cinnagen. Fetal bovine serum (FBS) (Cat # 10270) was purchased from GIBCO Invitrogen. Filter papers (Cat # DP 150‐150) and microplates (Cat # 3635) were purchased from Albet and corning, respectively. TSK G2000SWXL (Cat # 0008540), TSK G3000 SWXL (Cat # 0008541), and Guard (Cat # 0008543) were purchased from Tosoh. Quantikine ELISA kit for determination of G‐CSF (Cat # DCS50) was purchased from R&D systems.

### 2.1 Dendrimer synthesis

**FIGURE 1:**
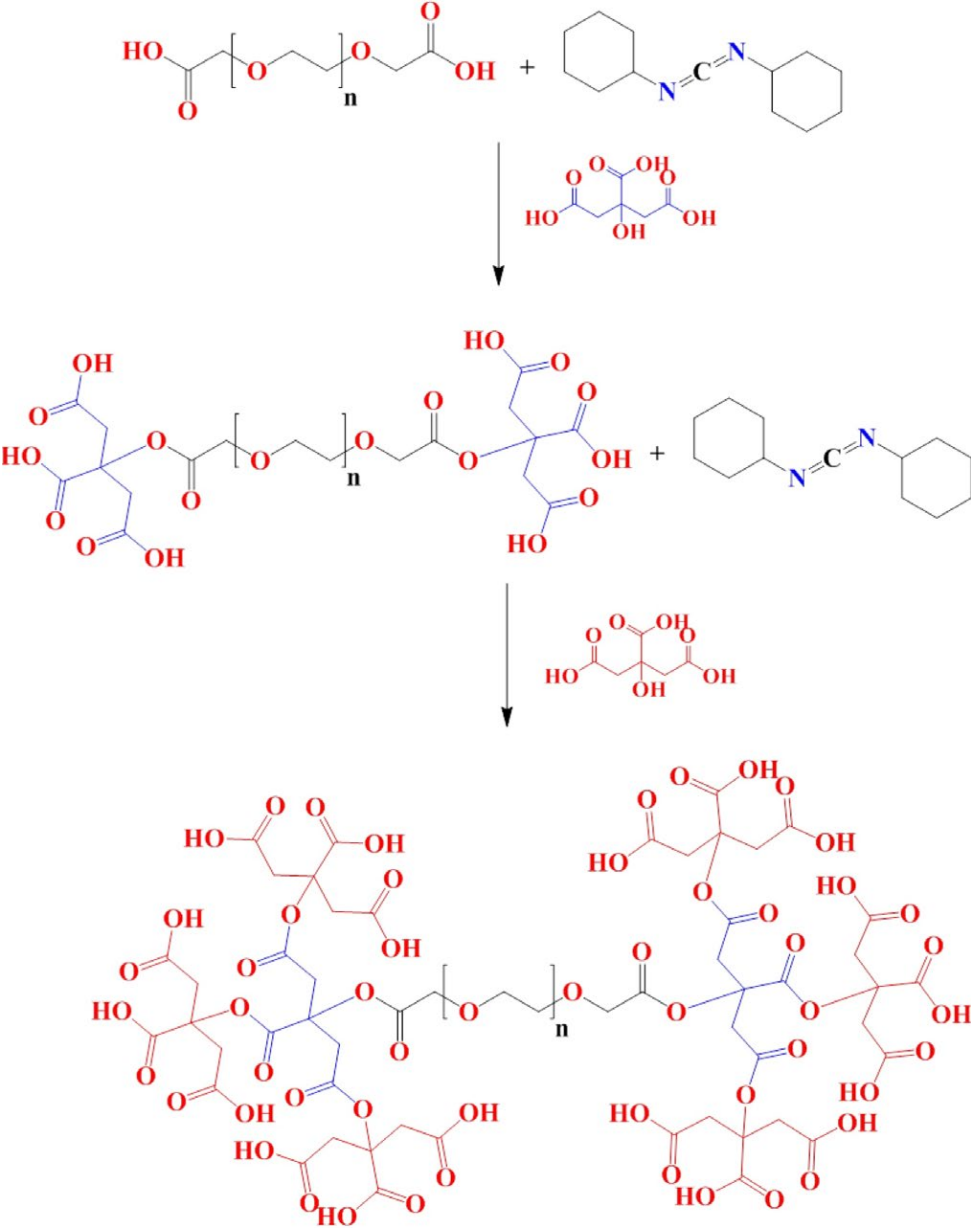
Mechanism of dendrimer synthesis using DCC as cross‐linker

To synthesize a negatively charged spherical linear dendrimer containing polyethylene glycol, the molar values accordance with the stoichiometric rules of DCC (0.812 g [3.95 mM]) and Poly (ethylene glycol) diacid (1 ml equivalent to 1.97 mM) were mixed in DMF (7.5 ml) for activation. After 10 min, citric acid (0.768 g equivalent to 3.95 mM) was added to react with the activated polyethylene glycol; after 3 h, the 15 ml of DCC (2.437 g equivalent to 11.85 mM) dissolved in DMF was added to activate the first generation of dendrimer, which was synthesized in the previous step; and after 10 min, the corresponding molar amounts of citric acid (2.304 g equivalent to 11.85 mM) were added and the reaction stirred for 1 day. Finally, to separate sediment particles and debris resulted from DCC, the resulting mixture was first diluted with DMF and passed through filter paper (DMF washed). The dendrimer was then purified using a column prepared from Sephadex G15. The steps of dendrimer synthesis are given in Figure 1.

### 2.2 Preparation of G‐CSF

To prevent possible interactions of components that exist in G‐CSF solution like acetic acid, G‐CSF was purified before the conjugation step. For this purpose, PD10 (G25) column was washed with 25 ml of 100 mM phosphate buffer (pH 7.4) and loaded with 2.5 ml G‐CSF containing solution. After leaving the 2.5 ml from the column, the next 3.5 ml was collected during the adding of extra above buffer.

### 2.3 G‐CSF dendrimer conjugation

**FIGURE 2:**
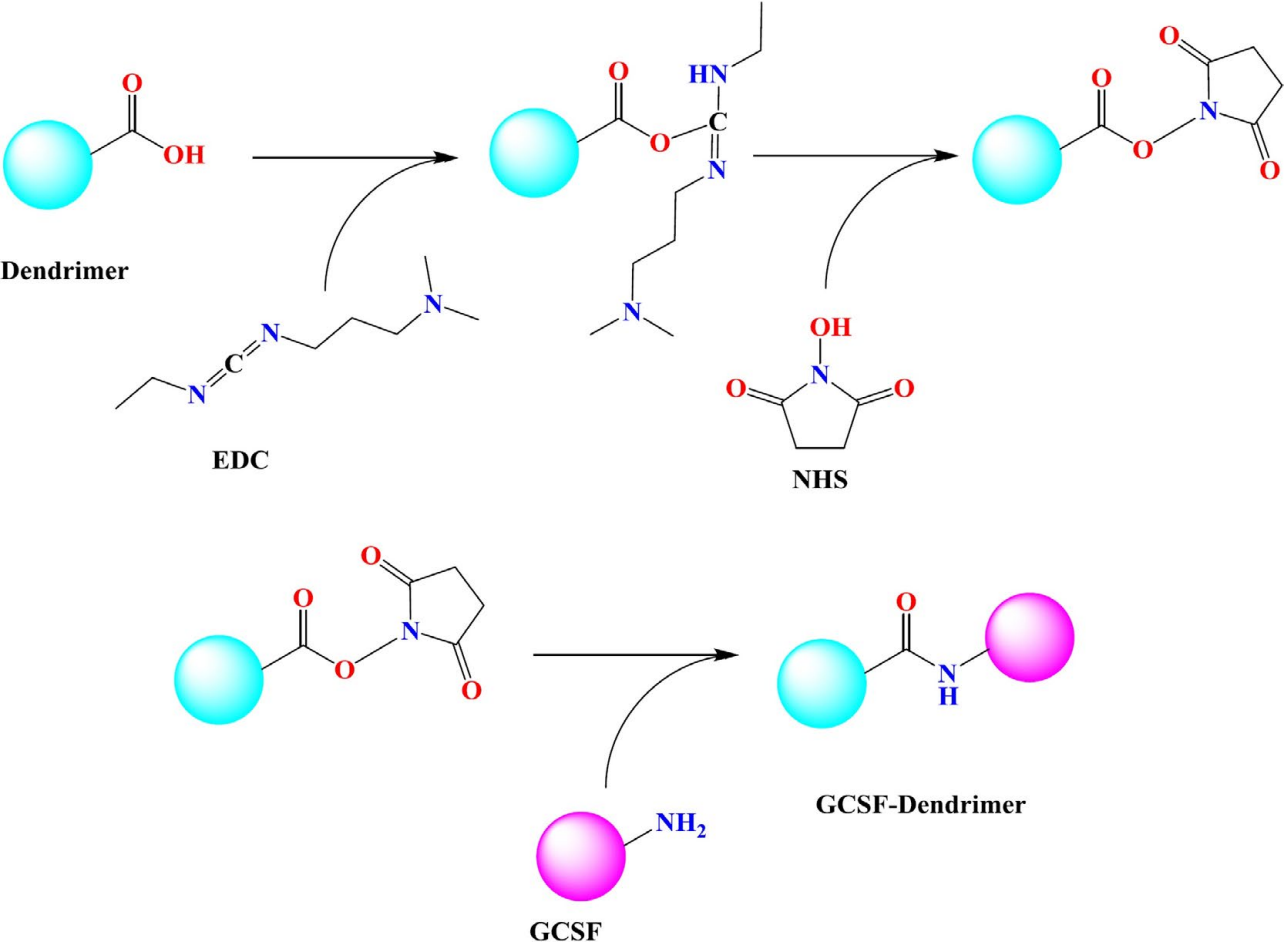
Mechanism of dendrimer conjugation to G‐CSF using EDC/NHS as cross‐linker

Activation was performed by EDC in the presence of NHS and DMF. For this purpose, 152 µl of dendrimer (40 mg/ml), 160 µl of EDC (20 mg/ml), and 88 µl of NHS (20 mg/ml) were mixed and incubated for 15 min at 120 rpm at room temperature on a magnetic stirrer. For the conjugation, 100 µl of the activated dendrimer was added drop wise to 3.2 ml G‐CSF (660 µg/ml) under mixing at 120 rpm on a shaker plate (activated G2: G‐CSF ratio of 6:1) for 2h at room temperature. The conjugation step was illustrated in Figure 2.

To purify the G‐CSF conjugate from other reactants, stop the conjugation reaction, and replace the reaction medium with an aqueous medium that maintains G‐CSF stability (10 mM acetate buffer, pH 4), PD10 column was used according to the instructions. First, the column was washed with 25 ml of the buffer and then 2.5 ml of a solution was loaded on it and after leaving the 2.5 ml from the column, the next 3.5 ml was collected during adding extra above buffer.

### 2.4 Conjugate characterizations

The morphology, mean size, and zeta potential were evaluated by atomic force microscopy (AFM) (Bruker, ICON) and dynamic light scattering (DLS) (Zetasizer Nano ZS; Malvern Instruments). Elemental analysis (Energy Dispersive Spectroscopy [EDS‐Map]) was done by field emission scanning electron microscope (FESEM, TESCAN). Fluorescence and ultraviolet‐visible (UV/Vis) spectroscopy were used to investigate conjugation using a fluorescence spectrophotometer (Perkin Elmer, LS 45) and UV spectrophotometer (Carry 100 Bio, Varian, Australia), respectively. In addition, Fourier‐transform infrared (FTIR) and biologic FTIR spectroscopies were done by FTIR spectrophotometer (AVATAR, Thermo) and Bio FTIR spectrophotometer (TENSOR II, Bruker), respectively.

### 2.5 Toxicity assays

A549 and L929 cells were used to evaluate the cytotoxicity of G‐CSF before and after conjugation. Briefly, different concentrations of the sample with a two‐fold serial dilution using the culture medium were prepared in 96 microplates. The cells (2.5 × 10^4^ cells/well) were seeded in RPMI 1640 medium supplemented with 10% FBS and incubated for 24 h at 37℃ and 5% CO_2_. After 48 h treatment with the compounds, XTT protocol was performed according to the instructions. The absorbance was read at a wavelength of 465 nm using a spectrophotometer and GEN5 software (Biotek XS2) was used for calculations.

### 2.6 In vitro biological activity

NFS60 cells were used to evaluate the biological activity of G‐CSF before and after conjugation on the growth and proliferation. Briefly, different concentrations of the sample with a twofold serial dilution using the culture medium were prepared in 96 microplates. The cells (3 × 10^4^ cells/well) were seeded in RPMI 1640 medium supplemented with 10% FBS, immediately treated with the serially diluted compounds, and incubated for 48 h at 37℃ and 5% CO_2_ (according to British Pharmacopoeia). Finally, XTT protocol was performed according to the instructions. The absorbance was read at a wavelength of 465 nm using a spectrophotometer (Biotek XS2) and GEN5 software was used for all calculations. Finally, the in vitro biological activity was measured and compared using Parallel Line Assay. For precise quantitation and better comparison, the tests were performed by using “G‐CSF international standard” from National Institute for Biological Standard and Control (NIBSC), which was approved by World Health Organization (WHO). In addition, Hoechst staining and Leica DMi8 microscope were used for cell imaging.

### 2.7 Polyacrylamide gel electrophoresis

The gels were prepared in non‐gradient (12, 15, and 20%) and gradient (10%–20%) states using the BioRad device and a slab gel (7 cm × 1 mm). Samples were prepared with a sample buffer containing sodium dodecyl sulfate (SDS) in both non‐reduce and reduce modes using 2‐Mercaptoethanol. Different staining methods including Coomassie Brilliant Blue, silver nitrate, and iodine (with barium chloride) were used for detection. To ensure the operation of the device and to investigate the separation of proteins with different molecular weights, a mixture of different pre‐stained molecular weights was used.

### 2.8 Size exclusion chromatography‐high performance liquid chromatography (SEC‐HPLC)

TSK G2000 and TSK G3000 SWXL columns (7.8 × 300 mm, column guard, Knauer HPLC device, Pump 1000 smart line, and Autosampler 3950 smart line) were used to quantify the small percentage of conjugates and to separate and change the molecular weight dispersion of each component in the reaction. The mobile phase was 10 mM ammonium carbonate buffer (pH = 7) with a flow rate of 0.5 ml/min (injection volume = 100 µl). A 2500 smart‐line UV Detector was used for detection at a wavelength of 215 nm and a running time of 25 min. The analysis of the results was done by EZ Chrome Elite software (Agilent).

### 2.9 In vivo biological activity

Briefly, male rats (200 ± 20 g) were used for in vivo biological test. Unconjugated and conjugated samples were prepared at different concentrations. IV injections were performed in the caudal region in groups (six rats per group). Blood samples were taken at different time points (2, 6, 24, 48, 72, 96 h after injection, and 1 day before injection) through a tail vein and mixed with EDTA. Cell count of WBCs was performed using a neobar slide and dilution with Marcano solution. Slides were prepared from the samples and after drying, Wright staining was used to differentiate the neutrophilic class under Nikon ELIPSE Ti microscope. Additionally, the blood samples were centrifuged, and the plasma was stored at −70℃ for determination of G‐CSF concentration. The G‐CSF concentrations were measured by ELISA assay kit at 450 nm using a spectrophotometer and GEN5 software (Biotek XS2). Finally, the terminal half‐life (*t*_1/2_) was determined using linear regression of the plasma concentrations as previously described.^20^

### 2.10 Abnormal toxicity

The test was performed according to British Pharmacopoeia as a standard protocol. Briefly, two healthy mice groups (*n* = 5, 17–24 g) received a slow intravenous injection of unconjugated and conjugated G‐CSF (0.5 ml/30 s). Then, mice were investigated within 24 h (and further 7 days) after injections. Moreover, the rat used for in vivo biological activity was also investigated within 7 days for body weight change and abnormal behavior.

### 2.11 Histopathological study

One week after a high‐dose injection of the samples into the rats, the animals were euthanized, and the vital organs (heart, kidney, and liver) were isolated and fixed in 10% formalin buffer. Tissue sections (5 µm in diameter) were prepared from the fixed tissues and stained with hematoxylin and eosin. The stained tissue slides were imaged using Olympus BX51 light microscope. Pathological changes such as acute and chronic reactions, necrosis, hemorrhage, as well as morphological changes were examined.

### 2.12 Biodistribution study

^99m^Tc radiolabeling was done based on Yang's study.^21^ Briefly, two groups of rats (five 250–300 g rats per each group) were anesthetized using sodium pentobarbital (40 mg/kg i.p.). Then, 250 μl of the solution including 200–300 MBq of ^99m^TcG‐CSF dendrimer was injected into the tail vein. Single‐photon emission computed tomography (SPECT) imaging was done at different time points of post‐injection (30, 60, and 120 min).^22^ After 120 min, the heart, liver, spleen, lungs, kidney, stomach, small intestine, large intestine, bone, muscle, and brain organs were collected and weighed. Radioactivity of each organ was measured using a γ‐counter, and the percentage of injected dose per gram of tissue was calculated.^23^

### 2.13 Statistical analysis

One‐way ANOVA post hoc test Tukey was used for data statistical analysis. Statistical analysis was performed using IBM^®^ SPSS Statistics software version 23 unless otherwise mentioned in the specified sections. All data are presented as mean ± standard deviation and a *p*‐value less than.05 was considered as a statistically significant difference, unless otherwise mentioned in the specified sections.

### 2.14 Ethics

All animal studies were conducted according to the ethical standards of the declaration of Helsinki. The ethical committee of Pasteur Institute of Iran has reviewed and approved the ethical considerations of the project.

### 2.15 Nomenclature of targets and ligands

Key protein targets and ligands in this article are hyperlinked to corresponding entries in http://www.guidetopharmacology.org, the common portal for data from the IUPHAR/BPS Guide to PHARMACOLOGY,^24^ and are permanently archived in the Concise Guide to PHARMACOLOGY 2019/20.^25^

## 3 RESULTS

### 3.1 Morphology, size distribution, and zeta potential

**FIGURE 3:**
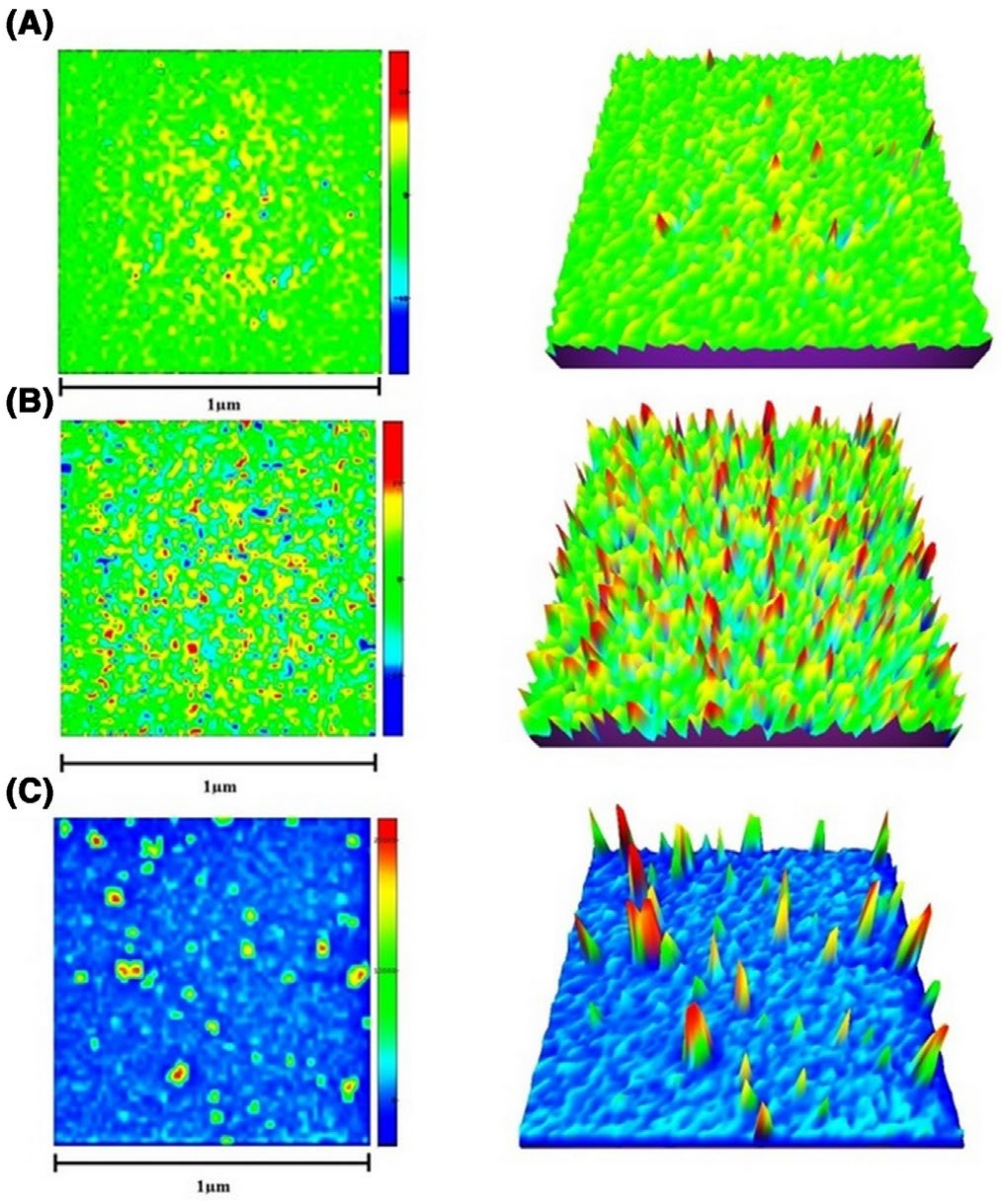
2D‐ and 3D‐AFM images of (A) dendrimer, (B) G‐CSF, and (C) G‐CSF dendrimer

**FIGURE 4:**
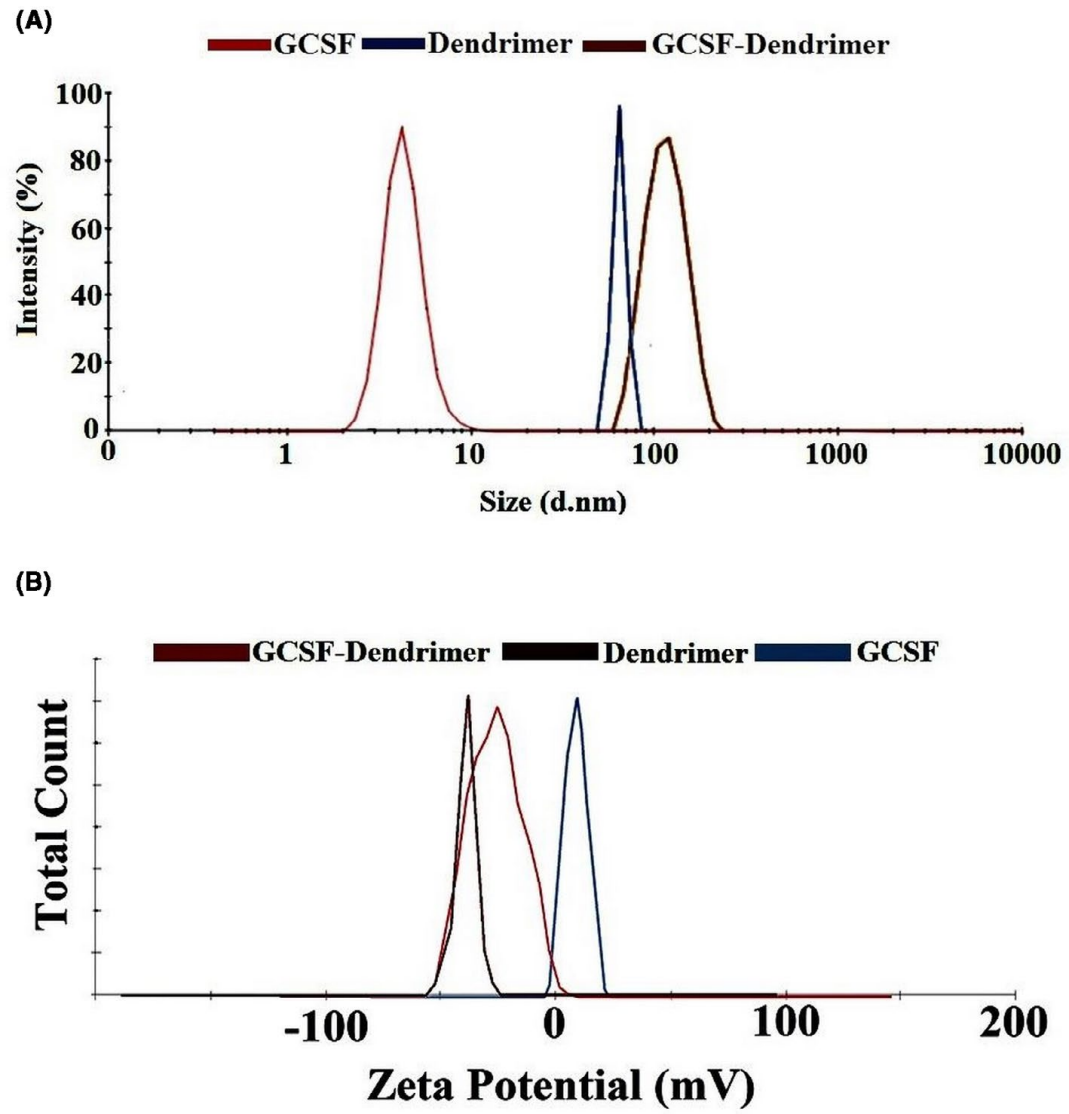
(A) DLS results: dendrimer (blue), G‐CSF (red), and G‐CSF dendrimer (brown); (B) zeta potential results: G‐CSF (blue), G‐CSF dendrimer (red), and dendrimer (brown)

Particle morphology and size distribution were examined using AFM analysis (Figure 3 and Figure S1). The particle size of G‐CSF, dendrimer, and G‐CSF dendrimer was measured as 7.12 ± 3.52 nm, 11.54 ± 3.57 nm, and 33.93 ± 7.5 nm, respectively. As can be seen, after conjugation, the particle size shows an increase that confirms the conjugation. The results were also confirmed by DLS analysis (Figure 4A). The difference in particle size by the two methods is related to the type of measurement and the sensitivity.^26^ The zeta potential of the conjugate was determined −17 meV in comparison to dendrimer (−28 eV) and G‐CSF (+8 eV) (Figure 4B).

### 3.2 Elemental analysis

**FIGURE 5:**
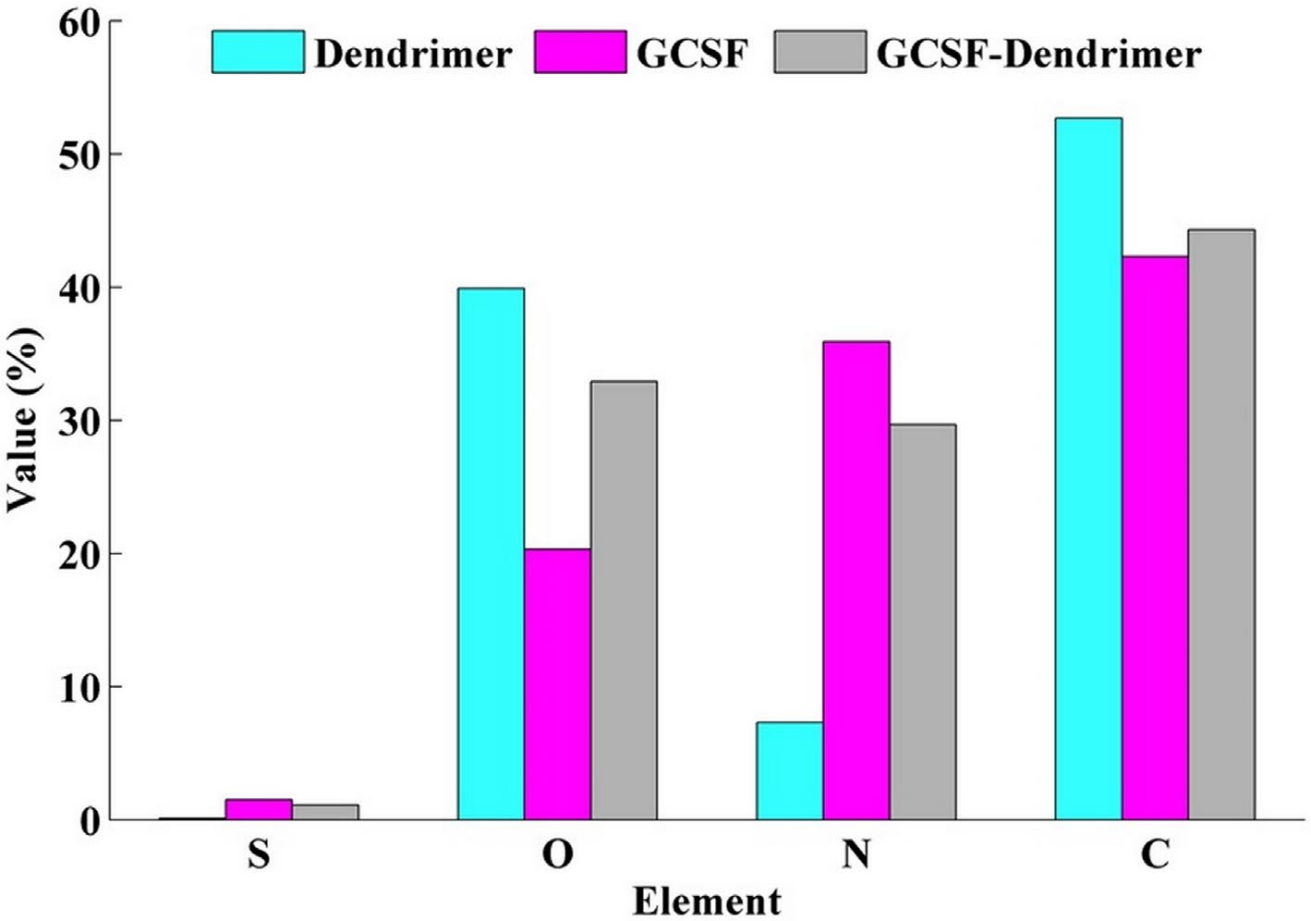
The percentage of elements in the structure of dendrimer (light blue), G‐CSF (purple), and G‐CSF dendrimer (gray). Data are represented as mean of triplicate measurements

Elemental analysis showed a significant increase in the amount of sulfur (changed from <0.1% to 1.1%, more than 10 times increase) and a relative decrease in the amount of carbon atom after the conjugation in dendrimer. In addition, the amount of oxygen in G‐CSF after conjugation was significantly higher than before (Figure 5 and Figures S2, S3).

### 3.3 FTIR

**FIGURE 6:**
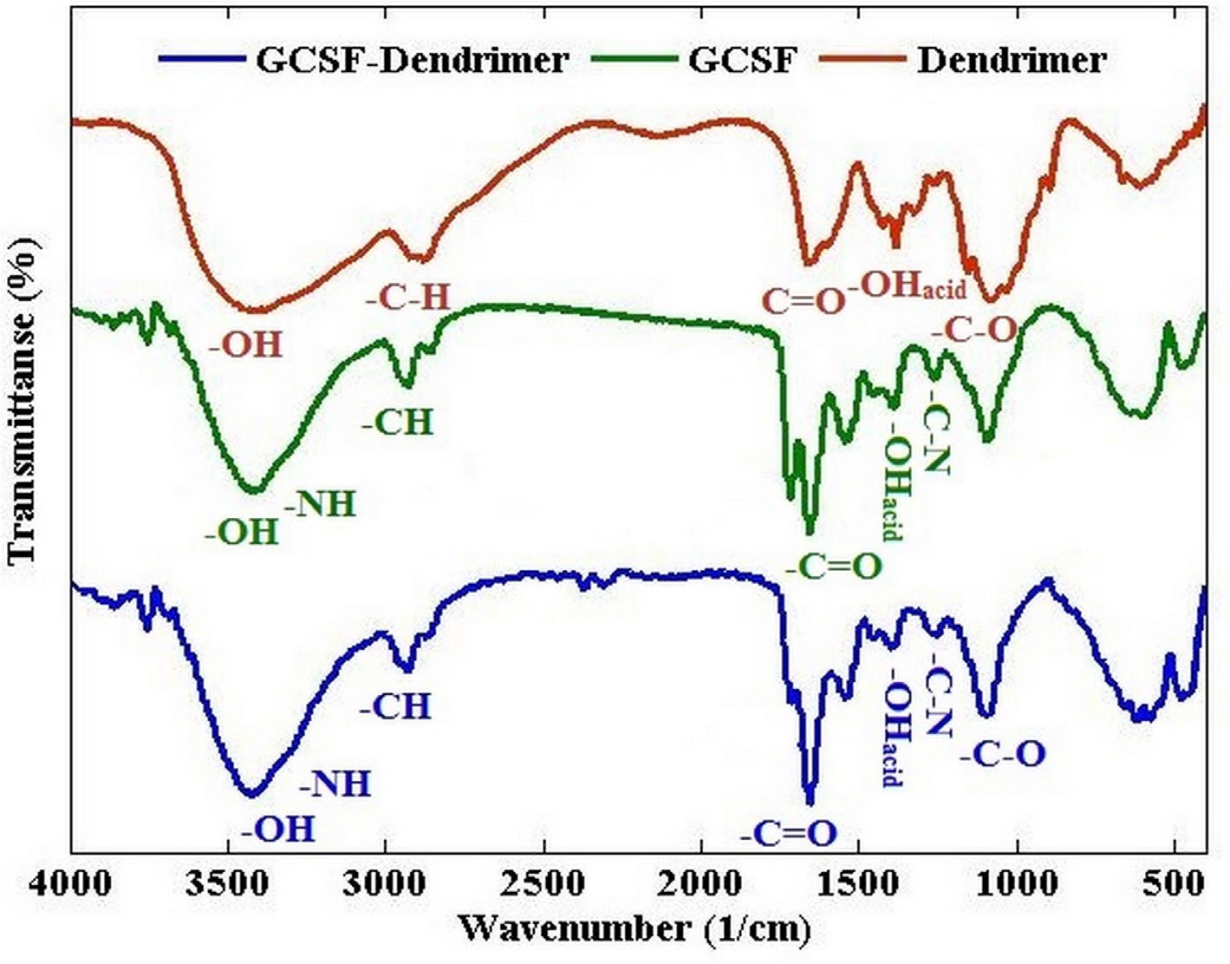
FTIR spectrum of dendrimer (red), G‐CSF (green), and G‐CSF dendrimer (blue)

**FIGURE 7:**
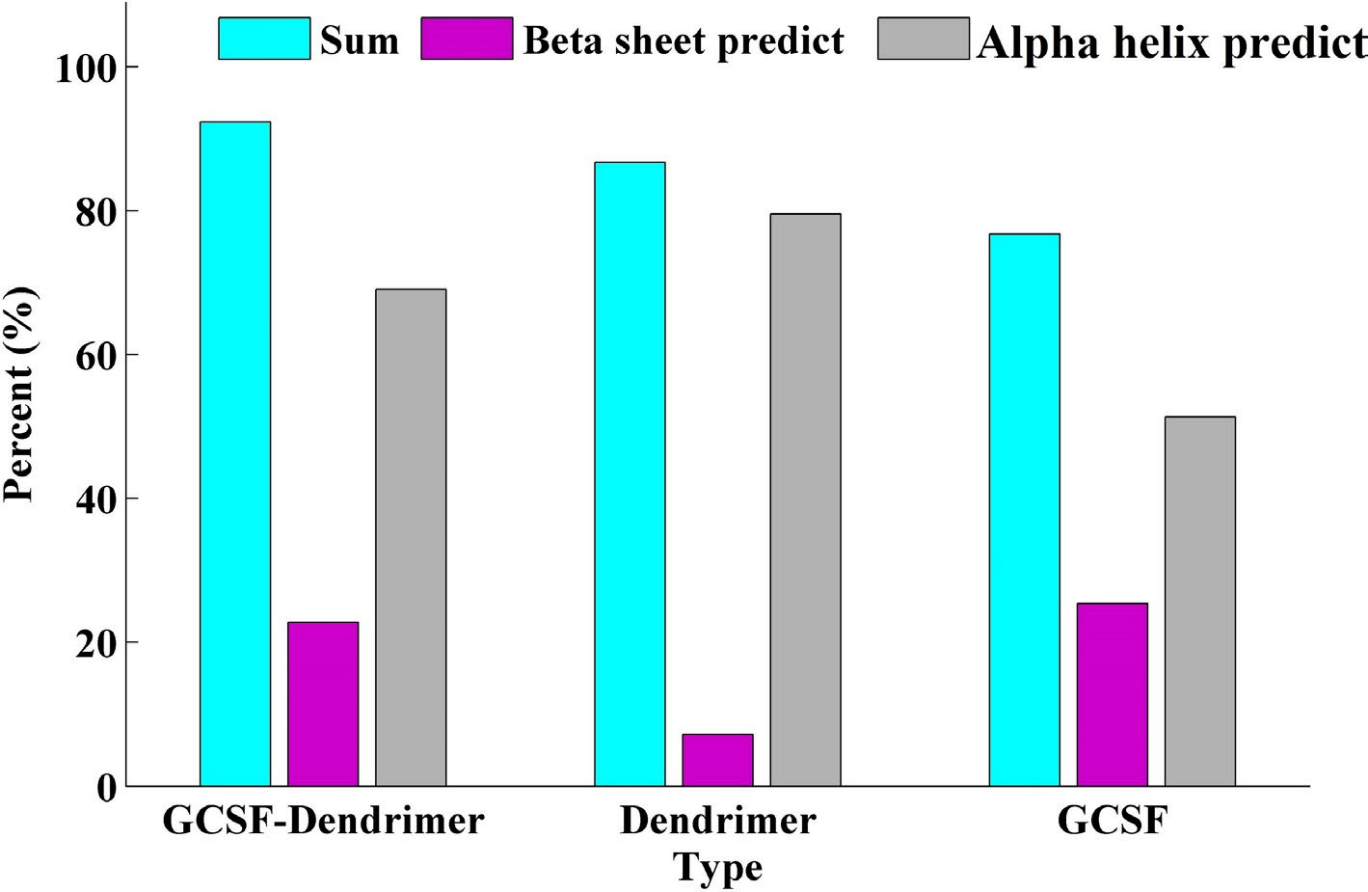
The predicted percentage of alpha‐helix (gray), beta‐sheet (purple), and sum (light blue) of the dendrimer, G‐CSF, and G‐CSF dendrimer. Data are represented as mean of triplicate measurements

The results of FTIR and biologic FTIR analysis are shown in Figures 6, 7, and Figure S4. Compared to the previous work, dendrimer synthesis was confirmed.^19^ By comparing the dendrimer and G‐CSF spectra with the final product, the shift created at the carbonyl peak (1710 cm^−1^) was the reason for the successful binding of G‐CSF to the dendrimer.

Biologic FTIR experiments revealed that the secondary structure of G‐CSF is different before and after conjugation. Data showed that conjugation causes changes in the bonds of molecule. As shown, the probability of alpha‐helix formation in the protein increases due to the dendrimer conjugation. Although there are slight changes in the beta‐sheet structures, the sum of two structures (alpha‐helix and beta‐sheet) is still different between before and after conjugation.

### 3.4 UV Spectrophotometry

As shown in Figures S5, G‐CSF shows two peaks at 230, 280 nm, and a deep valley at 250 nm. Comparison of the UV spectrum between conjugated and non‐conjugated states showed a decreased valley depth at 250 nm and, therefore, a decreased peak (280 nm)‐to‐valley (250 nm) ratio, which could be another indication of successful conjugation of protein to the dendrimer.

One of the characteristics of proteins is their ability to emit fluorescence (related to phenylalanine, tyrosine, and tryptophan residues in the structure), which can be attributed to their activities. As depicted in fluorescence spectra, G‐CSF has a maximum emission at a wavelength of 340 nm.^27^ After conjugation, the emission intensity decreased slightly and shifted to 350 nm, which confirms the conjugation and maintenance of its structure (Figure S6).

### 3.5 Polyacrylamide gel electrophoresis

**FIGURE 8:**
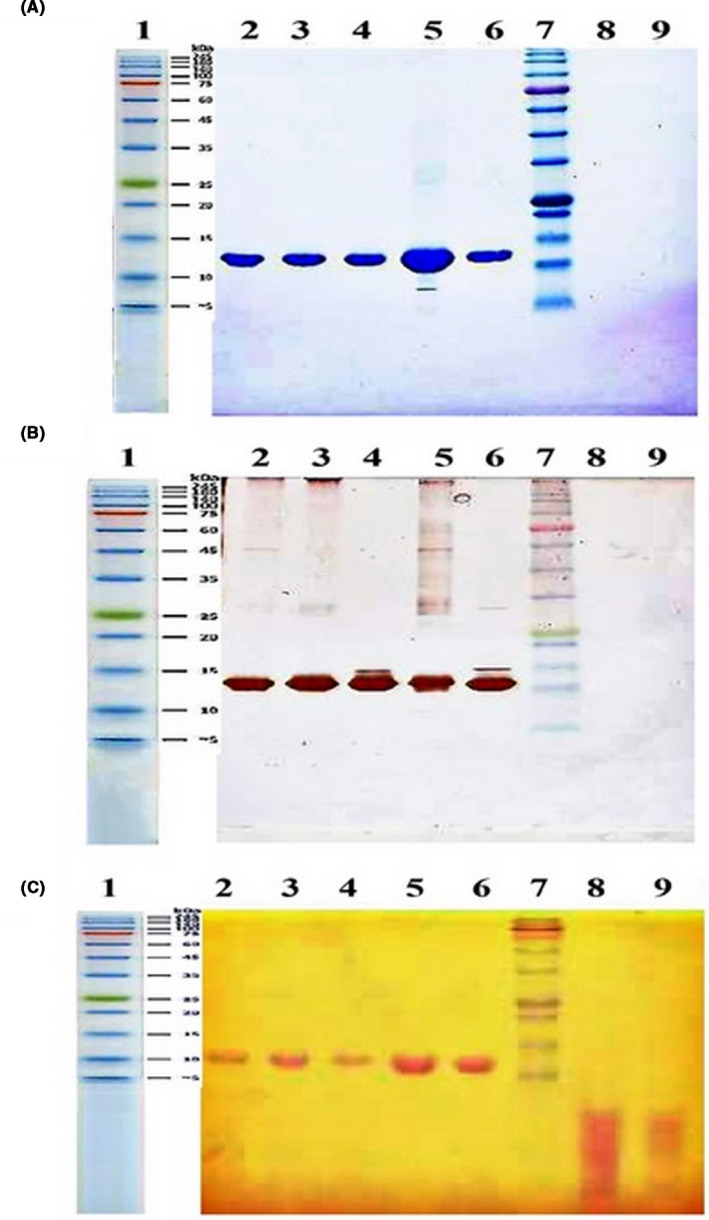
SDS‐polyacrylamide gel electrophoresis (SDS‐PAGE) images of conjugated G‐CSF (line 2–5), G‐CSF (line =6), marker (line =7), and dendrimer (line =8–9) using (A) Coomassie Brilliant Blue, (B) silver nitrate, and (C) barium chloride stains

The test performed at different conditions using different staining methods showed similar results. As shown in Figure 8, in Coomassie Blue and silver nitrate stains, dendrimer was not observed because it does not contain the protein structure. While the band related to the dendrimer was recognizable in the iodine staining method, there was no obvious difference in the electrophoretic mobility of the conjugated and non‐conjugated G‐CSF. This could be due to the low sensitivity of the test to separate the closed molecular weights or even due to an increase in the molecular velocity of the molecule after condensation with the dendrimer.

### 3.6 SEC‐HPLC

**FIGURE 9:**
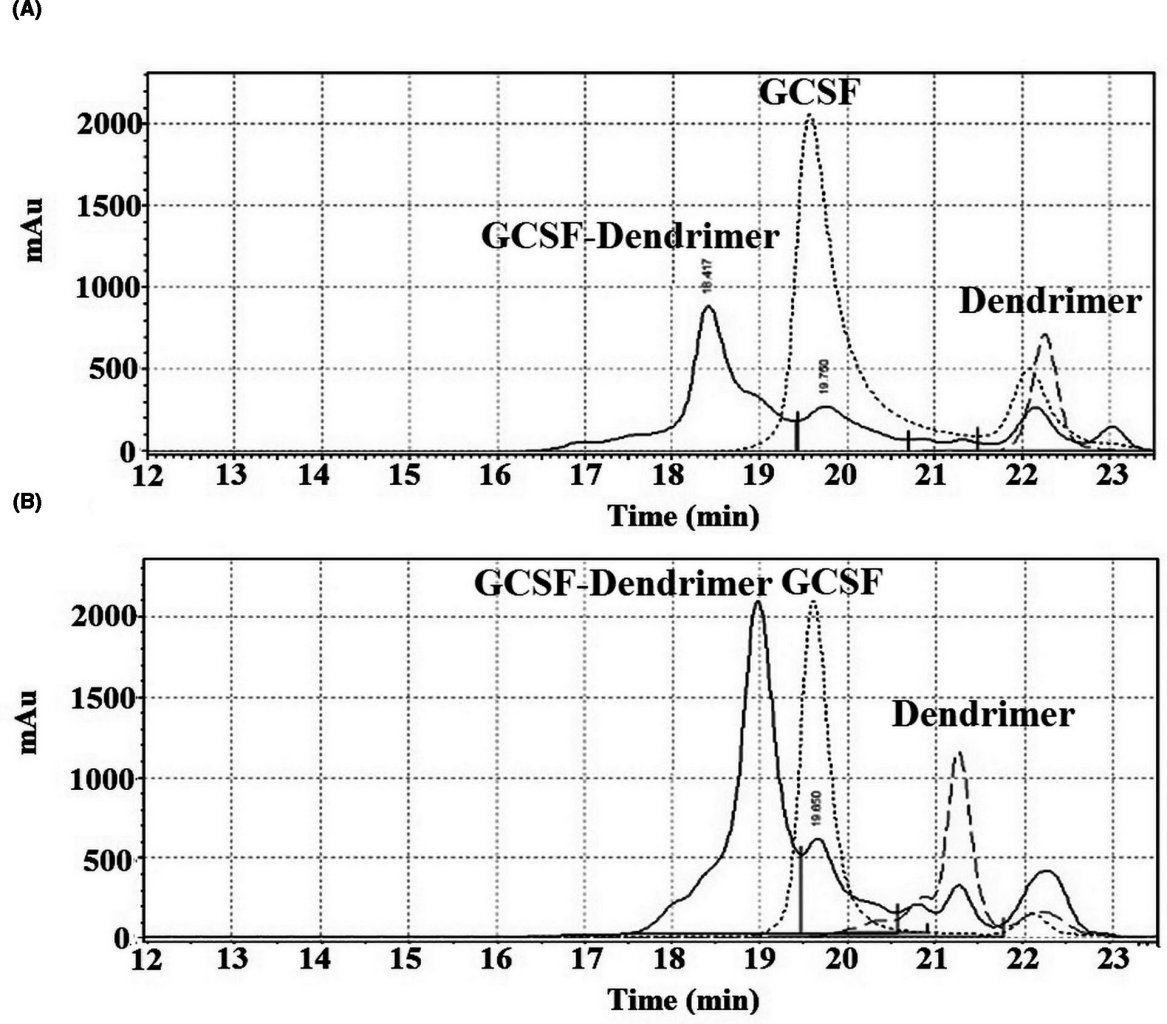
SEC‐HPLC chromatogram of G‐CSF (black dot), dendrimer (Green dash), and G‐CSF dendrimer (Blue line) using (A) swxl G2000 and (B) swxl G3000 column

As shown in Figure 9, the retention time of G‐CSF is about 19.7 min, which changes to 18.4 and 19.0 min in TSK G2000 and TSK G3000 SWXL columns after conjugation, respectively. Changing the retention time also confirmed the conjugation.

### 3.7 Cytotoxicity

**FIGURE 10:**
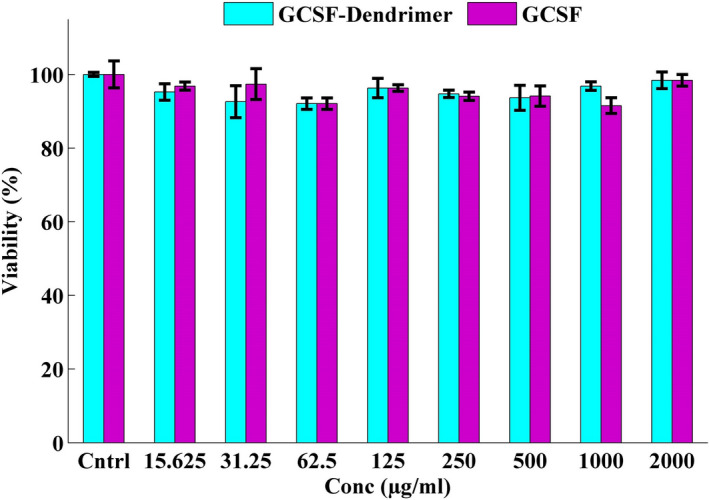
XTT assay results of G‐CSF (purple) and G‐CSF dendrimer (blue) on A549 cell lines after 48 h treatment. Data are represented as mean ± standard deviation from four independent replicates

The toxicity of G‐CSF and G‐CSF dendrimer on both cells (human‐originated [A549] and the mouse‐originated cells [L929]) was evaluated and compared with each other as well as the control group. The data elucidated that none of the compounds is toxic and there was no significant difference between the conjugated and non‐conjugated protein (Figures S7, S8, and Figure 10).

### 3.8 In vitro biological activity

**FIGURE 11:**
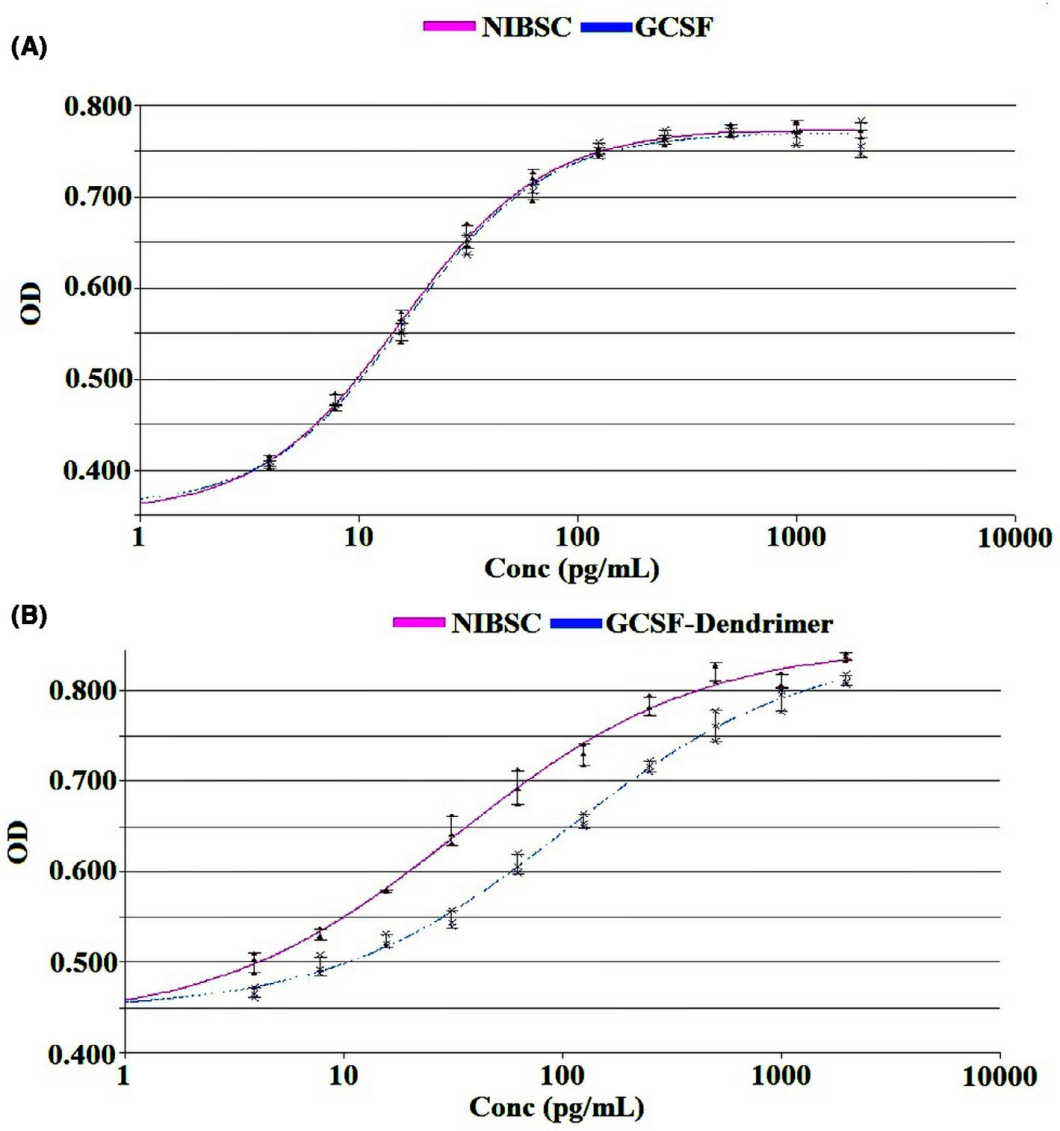
In vitro biological activity results by XTT assay of (A) G‐CSF and (B) G‐CSF dendrimer on NFS60 cell lines after 48 h. Data are represented as mean ± standard deviation from four independent replicates

**FIGURE 12:**
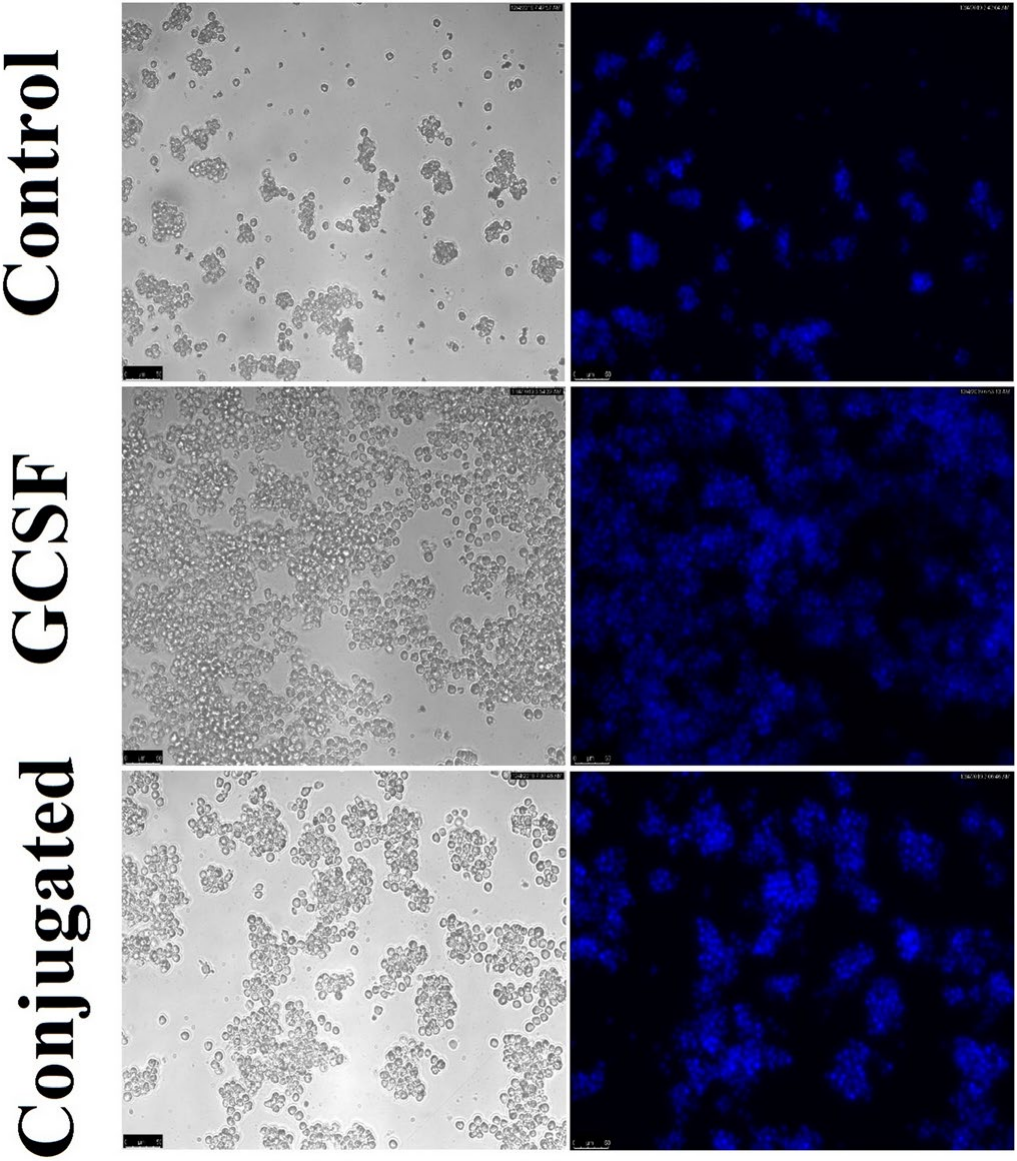
Inverted microscope (left) and fluorescence (right) images (NFS60) using Hoechst stain of G‐CSF and G‐CSF dendrimer (100×)

The results indicated that G‐CSF is active in both pre‐ and post‐conjugation states, and causes cell proliferation and growth compared to the control group. However, the biological activity drops sharply from 96% (equivalent to 96 × 10^6^ IU/mg) to 34% (equivalent to 34 × 10^6^ IU/mg) after the conjugation (Figures 11, 12, and Figure S9).

### 3.9 In vivo biological activity

**FIGURE 13:**
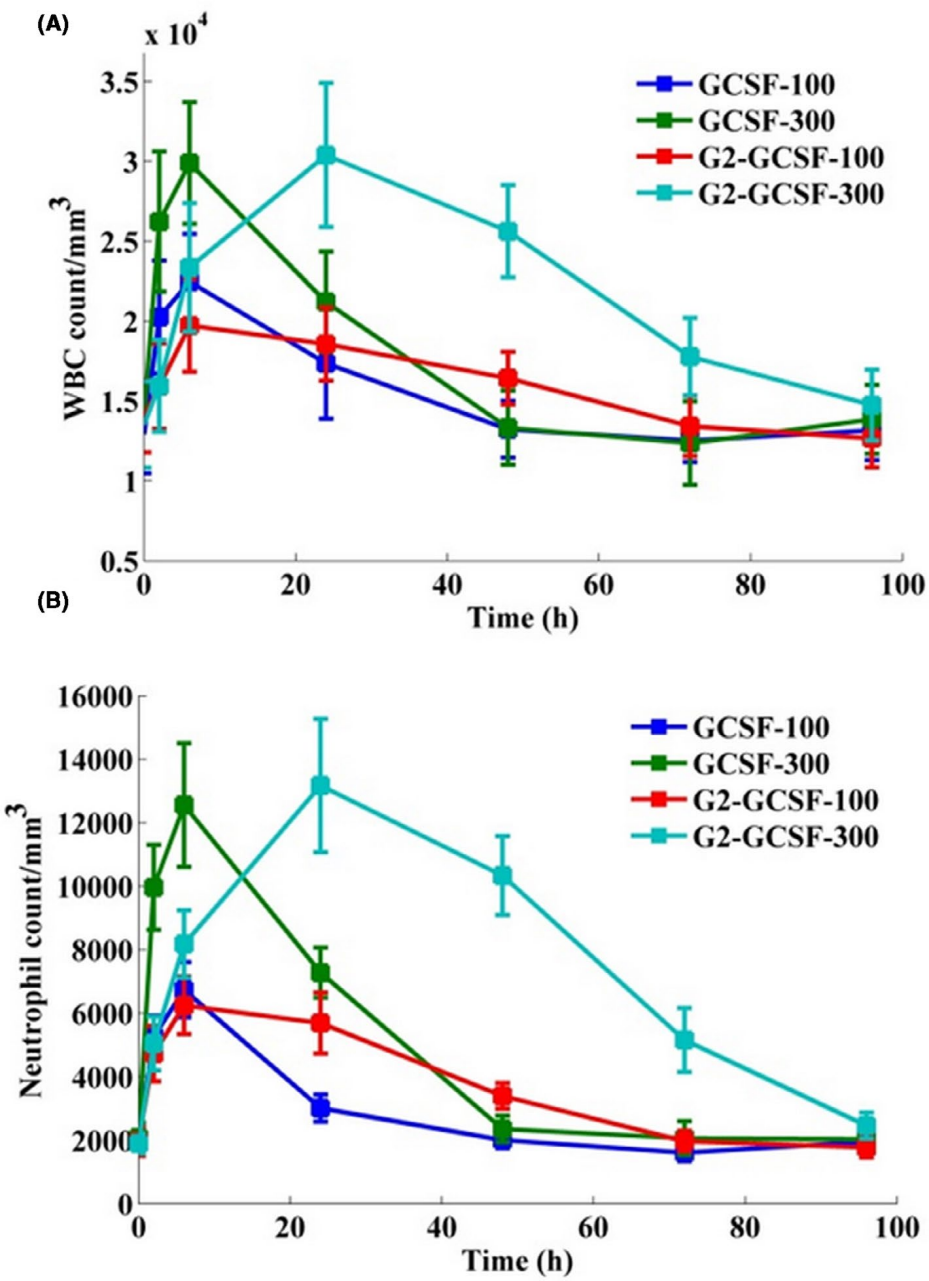
In vivo biological activity results (100 and 300 µg/kg). (A) White blood cell and (B) neutrophil profile at different times. Data are represented as mean ± standard deviation from six independent replicates

**FIGURE 14:**
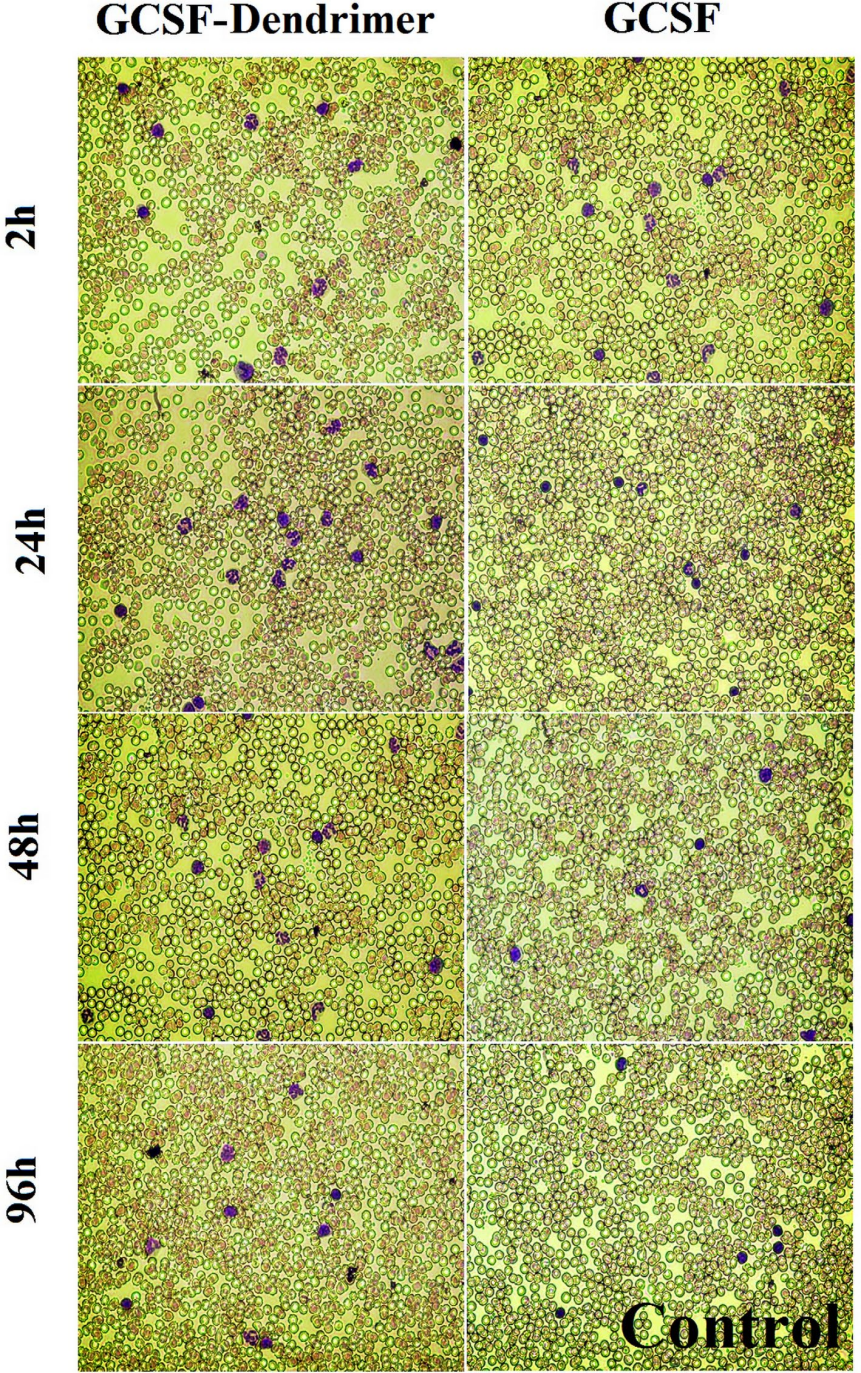
200× images related to peripheral blood slide—300 µg/kg of G‐CSF and G‐CSF dendrimer at different times (WBC = purple nucleated cell)

As shown in Figures 13 and 14, not only the conjugate did not show a decrease in the activity but also showed an increase in the activity. Although the enhanced activity was not visible early (0–6 h), and to some extent, the free form shows more activity. However, after 6 h, and especially at 24 h, this increment was obvious. Moreover, it turns out that the biological activity is maintained for a longer time period (at least up to 48 h), and the retained biological activity increases slightly with increasing the dose up to 72 h. The white blood cell counts dropped sharply after injection of 100 µg/kg G‐CSF in 24 h and 300 µg/kg G‐CSF in 48 h and reached near the initial level before the injection. However, in the conjugated form, these times were 60 h and 96 h for a dose of 100 µg/kg and 300 µg/kg, respectively. In other words, to achieve an increase in white blood cells and maintain a higher count than the initial limit, daily injections were required for the free form; while in the conjugated form, injection intervals of 3 or even 4 days are enough. Moreover, to check the ability of G‐CSF in the enhancement of body immune cells, the neutrophil count was examined after the injections. For example, at peak time, the conjugated form caused doubles of the total white blood cell count (compared to before the injection) but has the potential to increase neutrophil counts to 6.5 times. The neutrophil count increased by 250% and 10% after 24 and 48 h of injection of 300 µg/kg G‐CSF, respectively. But the neutrophil count increased 550% and 400% at the same time for the conjugated form. The area under the curve of the conjugate was 2.5‐fold greater than the non‐conjugated form for 96h of post‐injection.

Additionally, the mean of plasma G‐CSF concentration was measured 2456 ng/ml and 800 pg/ml after 2 and 24 h G‐CSF injection (300 µg/kg), respectively, and then was not detectable. While, the concentration was, respectively, 3291, 33 ng/ml, and 280 pg/ml for 2, 24, and 48 h after conjugate injection (300 µg/kg), and then was not detectable. The terminal half‐life (*t*_1/2_) was calculated at 2 and 3.5 h for free and conjugated G‐CSF, respectively.

### 3.10 Abnormal toxicity

Abnormal toxicity of G‐CSF and G‐CSF dendrimer was investigated according to British Pharmacopoeia in mice. In both groups, none of the mice died and the test was passed. Additionally, there was no noticeable difference between the conjugated and non‐conjugated protein for a mean of body weight (231 ± 13 and 227 ± 16 g, respectively) and/or any abnormal behavior in rats.

### 3.11 Histopathological study

**FIGURE 15:**
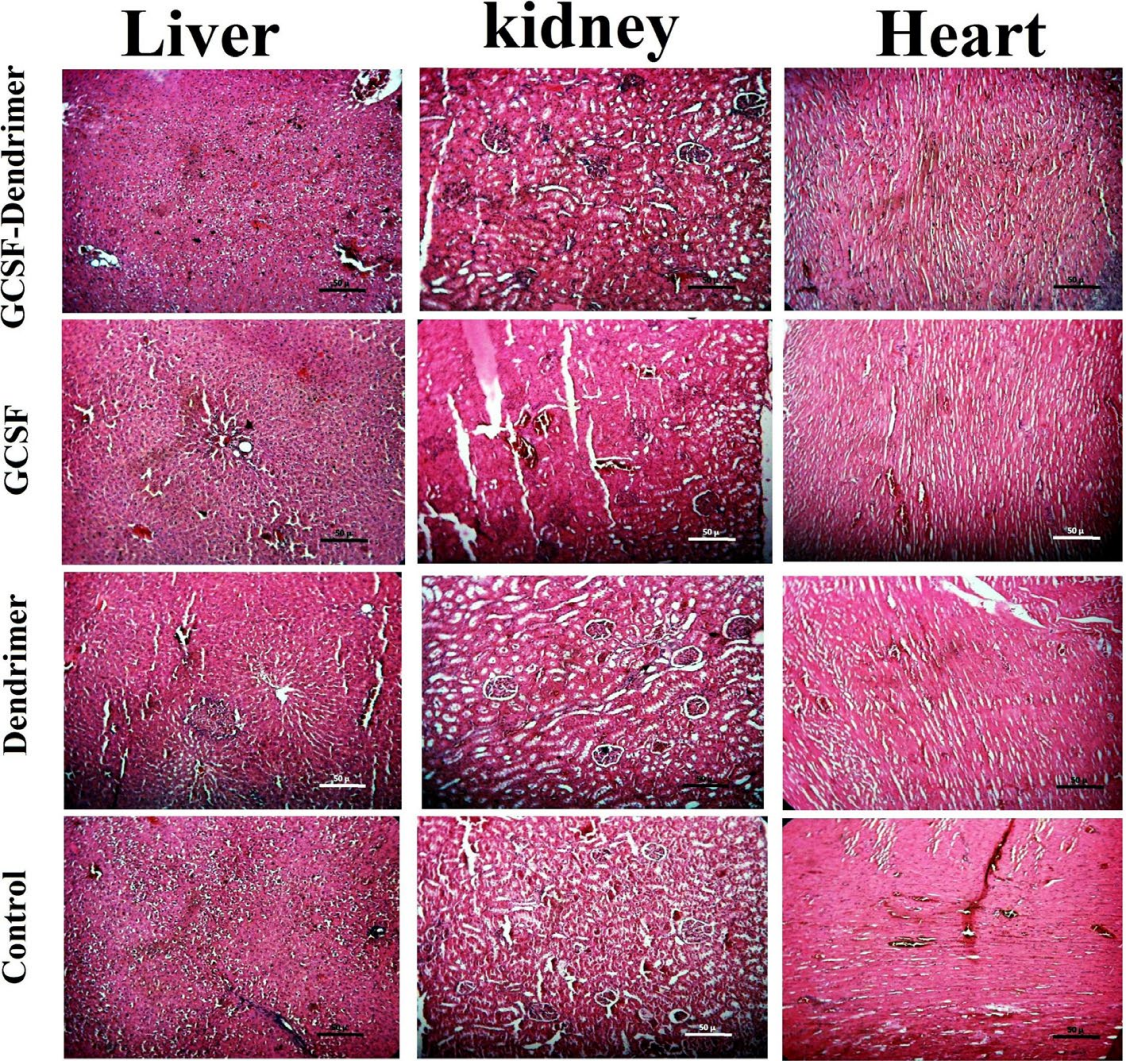
Histopathological sections of liver, kidney, and heart in control, dendrimer, G‐CSF, and G‐CSF dendrimer using H&E stain (100× images)

As shown in Figure 15, despite the use of higher doses of conjugated G‐CSF, no pathological adverse effect was observed in the vital organs (heart, liver, and kidney), and the tissues were microscopically similar to the unconjugated protein. Both tissues were equivalent to the control group, but with a small amount of hyperemia possibly due to the G‐CSF function on endothelial cells and vascularity. The dendrimer also did not show pathological effects.

### 3.12 Biodistribution

**FIGURE 16:**
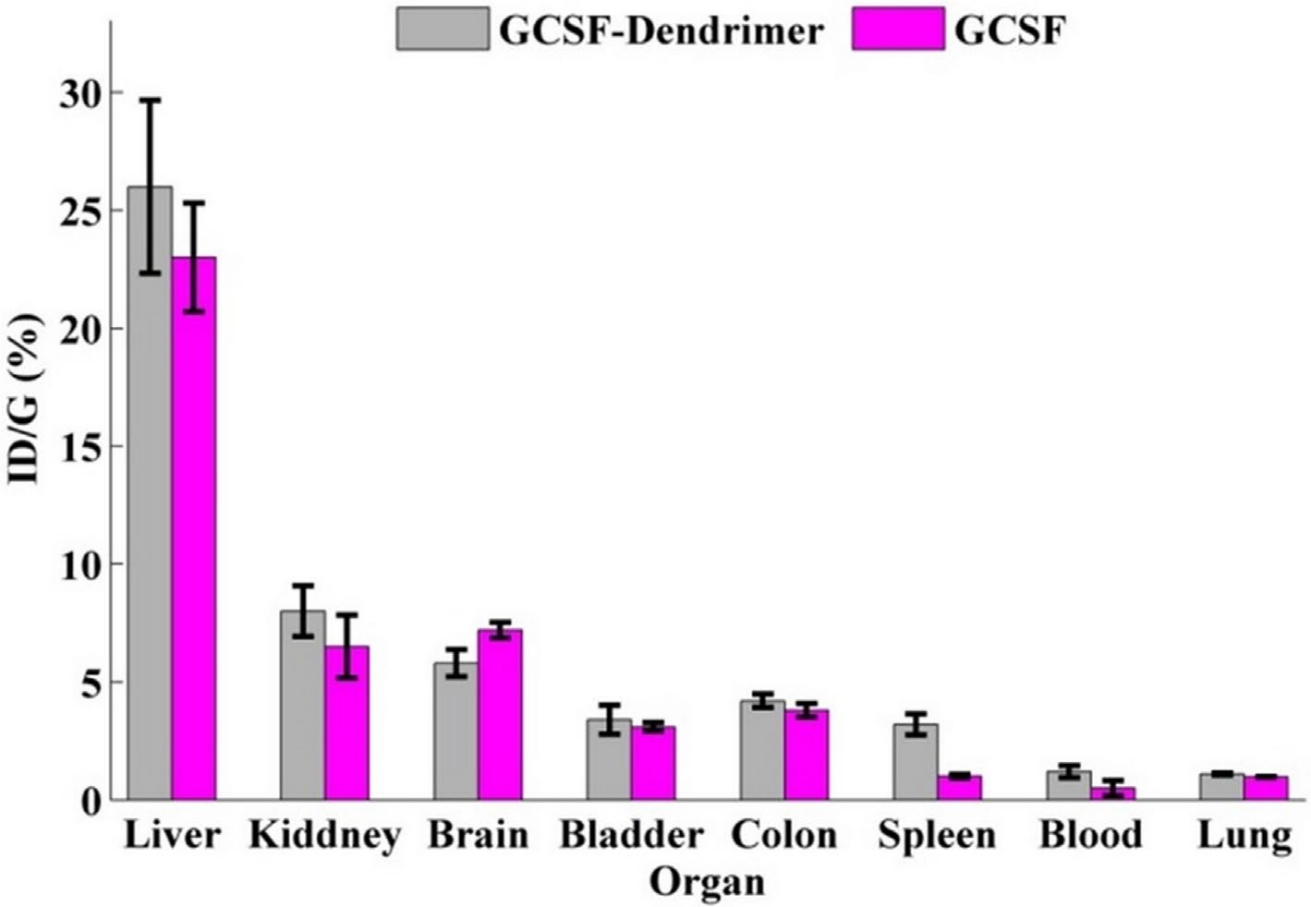
Biodistribution results obtained from SPECT images of G‐CSF (purple) and G‐CSF dendrimer (gray). Data are represented as mean ± standard deviation from five independent replicates

Imaging results at different times showed that the biodistribution of conjugate is similar to G‐CSF. Also, a comparison of biodistribution after 120 min showed that the highest distribution was in the liver, kidney, and brain tissues (Figure 16 and Figure S10).

## 4 DISCUSSION

In this study, the effect of anionic globular dendrimer was investigated on G‐CSF, especially its potency. For this purpose, after dendrimer synthesis, the activation was performed in the presence of EDC, NHS, and DMF and the conjugation reaction was finally completed in a phosphate buffer medium. Since any technique may have problems, limitations, or errors, so in this research, various test methods were used to cover the limit of one test with a test or other tests. Different techniques including in vitro and in vivo biological tests, as well as nano‐based characterizations employed to confirm the conjugation between dendrimer and G‐CSF. Some of the experiments just focused on the change in nanoparticle or protein properties, while some of them were applicable for both. Finally, we verified the conjugation step by comparing data obtained from these tests.

As expected, the conjugate should have different physicochemical properties in terms of size, charge, number of elements, UV spectra, and secondary structure. Therefore, these properties were assessed by different techniques to confirm the conjugation between dendrimer and G‐CSF molecule. DLS data elucidated an increase in the hydrodynamic volume of the conjugated molecule. SEC‐HPLC revealed an increase in the molecular weight of conjugate. Atomic force microscopy also confirmed the increase in the molecular size as previously showed by SEC‐HPLC. As expected, the zeta potential of conjugate became more negative than that of non‐conjugated G‐CSF. The result of EDS‐Map showed a relative increase in sulfur and oxygen atoms after the conjugation relative to free dendrimer and free G‐CSF, respectively. FTIR illustrated a slightly different pattern compared to the native protein. FTIR analysis in an aqueous environment (that is most important for protein molecules) showed an increase in the alpha‐helix structure after the conjugation step. Finally, the change in the pattern of UV spectrum also indicates the attachment of G‐CSF to the dendrimer structure.

The toxicity of compounds was studied both in vitro and in vivo. The results obtained from the in vitro test revealed that there was no significant difference between G‐CSF cytotoxicity before and after conjugation, and no difference with the control group. In addition, the toxicity pattern on the mouse‐ and human‐originated cells are similar and there is no noticeable difference between them. The results of the in vivo test also elucidated that the conjugated and non‐conjugated G‐CSF molecules have no specific pathological effect on the vital organs such as heart, liver, and kidney after a high‐dose administration to the rats. Moreover, the toxicity test did not show any abnormal toxicity for both G‐CSF forms in mice.

The most important difference was observed between the results of biological activity in vitro and in vivo for the conjugate. In vitro biological test showed that G‐CSF is active before and after conjugation and could stimulate cell proliferation and growth of cells. However, the in vitro biological activity declined one‐third after the conjugation (relative to free form) as observed in the previous studies like G‐CSF PEGylation.^10^ Nonetheless, not only conjugate did not show a decrease in vivo biological activity but also showed an increase in the activity. In other words, conjugated G‐CSF has more capacity to increase white blood cell count, mainly due to the sharp and consistent increase in neutrophil count. This phenomenon may be explained by enhanced plasma half‐life of the conjugate similar to PEGylated or albumin‐fused forms^10^, ^28^ or sustained release of the drug, long‐time G‐CSFR activation, and delay deactivation in cells that need future investigations.

Since 2000, nanoparticles have been gradually considered in G‐CSF‐related researches. However, most of these investigations have been performed for sustained release using loading techniques. Over time, little research has been done on the conjugation of G‐CSF with nanoparticles like conjugation with Heparosan. In one of the first works done by Ueno et al., the loading technique and calcium carbonate nanoparticles were used. They just assessed the in vitro release of G‐CSF from the nanoparticles and no study was performed in terms of in vitro or in vivo biological activity and toxicity.^29^ Research by Choi and Park used PLGA nanoparticles, and similarly, their study lacked biological activity and toxicity tests.^30^ Apart from the type of nanoparticles they used, one reason for such incomplete works can be based on the fact that more than 80% of release occurred in less than 48 h. Likely, this release occurs much more rapidly in the physiological environment, where the conditions are more complex than the in vitro tests. Therefore, the time of in vivo release will be very short and insufficient for such assessments. Subsequent research has shown that the use of PLGA alone increases the adsorption of protein to the PLGA matrix, which ultimately leads to an incomplete protein release. Liu and Yuan established a new formulation by loading the G‐CSF molecule on dextran nanoparticles.^31^ They sprayed this formulation on a scaffold, then coated it with PLGA, and finally added free G‐CSF. The in vivo studies showed that the biological activity of this formulation was maintained for a longer period than previous studies; but they had two issues with such a formulation. First, they used two types of nanoparticles in the formulation that resulted in a complex formulation in the preparation step, and the second, the difference between the stability of free and adsorbed G‐CSF molecules in the pharmaceutical dosage forms that restrict the clinical application and route of administration. Liu and Yuan attempted to modify their study. After loading the G‐CSF molecule on the dextran nanoparticles, they proceeded to encapsulate the dextran nanoparticles containing G‐CSF by PLGA, which eventually led to the formation of a microsphere.^32^ However, they were not able to solve the mentioned problems, even by such modifications, the final particle size increased significantly and reached 100 µm. Therefore, in this study, we tried to maintain the biological activity of G‐CSF for a longer period by using only one type of nanoparticle (that conjugated to G‐CSF molecule), and solve the restriction of clinical application, simultaneously. It should be noted that Liu and Yuan reached to threefold increment in the biological activity in term of increase in the neutrophil count, while in our study this increase was 5.5‐fold.

In conclusion, in the current study, for the first time, an anionic globular dendrimer was used as a nanoparticle to conjugate G‐CSF to enhance the in vivo activity. Our data suggest that dendrimers could improve and enhance the G‐CSF biological activity despite a decrease in the in vitro activity. However, the exact mechanism is still unclear and other assessments like signaling pathways or downstream gene expression analysis should be done in future studies. Initial toxicity tests in the present study elucidated that the conjugate did not show severe cyto‐ and/or tissue toxicities in the animal model. However, since G‐CSF has some side effects, such as fever and back pain, further investigations should be conducted in the field of pharmacology, because the developed conjugate has a higher residence time in the body. Unfortunately, we could not measure some bystander effects such as fever because our sample preparations were not carried out under GMP and non‐pyrogenic conditions; and therefore, any increase in the temperature could not specify the cause of fever including the nature of the drug or the process challenges like endotoxin. Moreover, it may be possible to use the multivalent capability of the dendrimer for targeting purposes (such as stem cell therapy, cancer targeting, and muscle and tissue repairing) or other new applications that could be incorporated and considered in future studies.

## DISCLOSURE

The authors declare there is no conflict of interest.

## AUTHOR CONTRIBUTIONS

MSA and RAC developed the idea and designed the experiments. SSMM, SS, MS, and VM conducted the experiments. MSA, RAC, KA, and BV analyzed the data. SSMM wrote the manuscript. All authors confirmed the final manuscript before submission.

## Supporting information

Figure S1

Figure S2

Figure S3

Figure S4

Figure S5

Figure S6

Figure S7

Figure S8

Figure S9

Figure S10

Supplementary Material

## Data Availability

The data that support the results are available from the corresponding author upon reasonable requests.
