# Supplementary figures and images for: Novel G‐CSF conjugated anionic globular dendrimer: Preparation and biological activity assessment
Source: Pharmacol Res Perspect. 2021 Jul 16;9(4):e00826. doi: 10.1002/prp2.826 (PMC8283867; doi:10.1002/prp2.826)

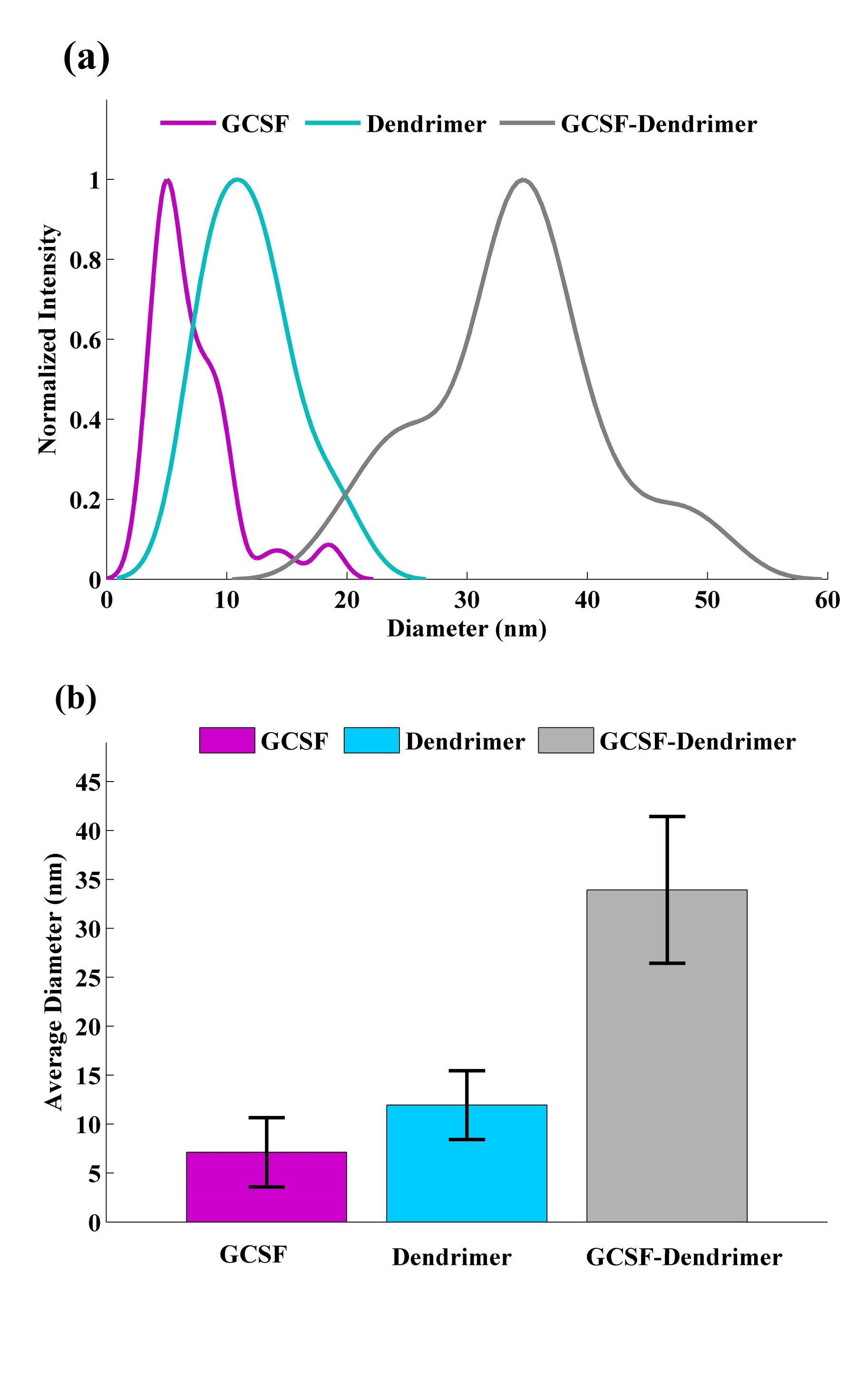

Supplement: Supplementary file 1 — Figure S1 [file PRP2-9-e00826-s008.jpg]

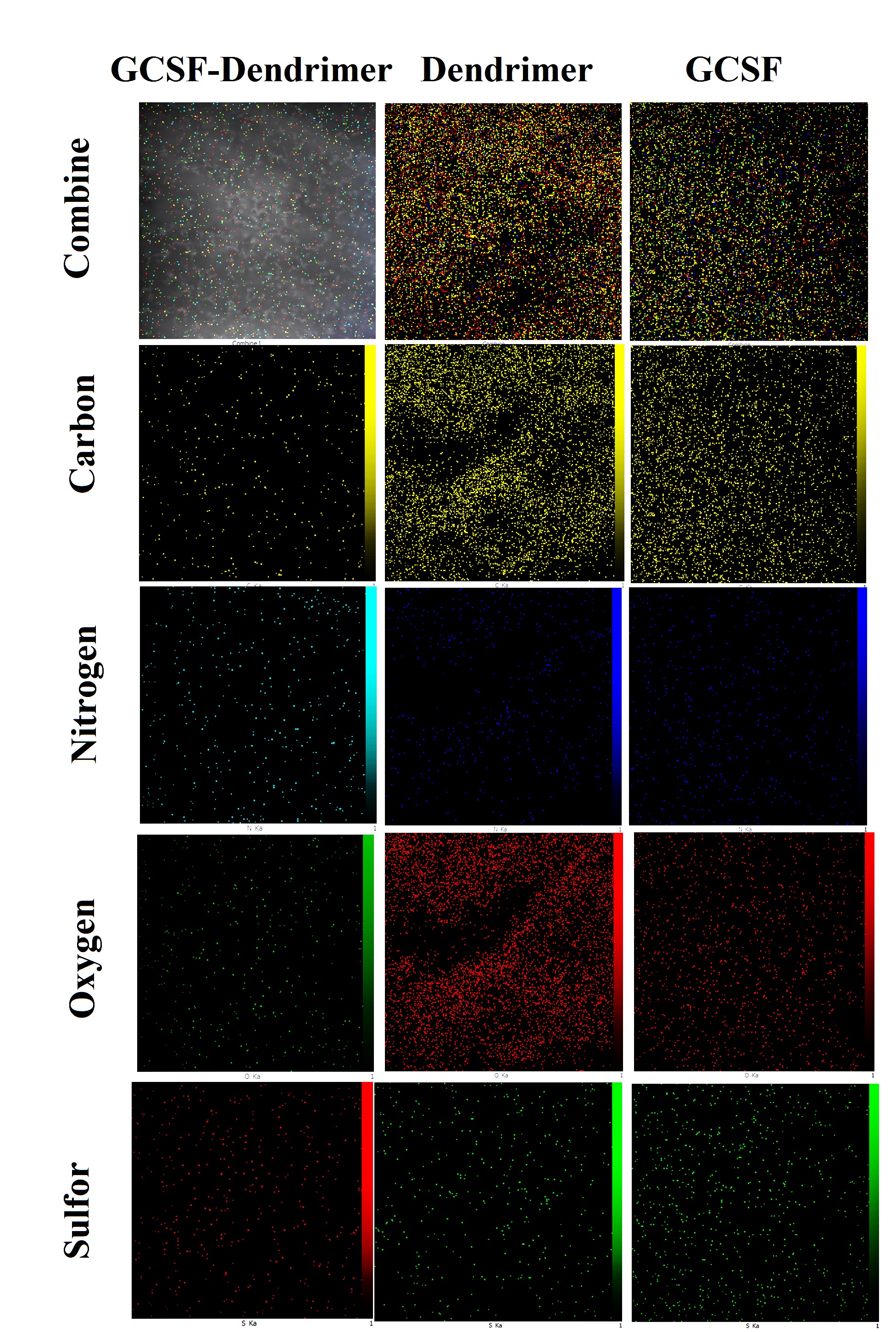

Supplement: Supplementary file 2 — Figure S2 [file PRP2-9-e00826-s010.jpg]

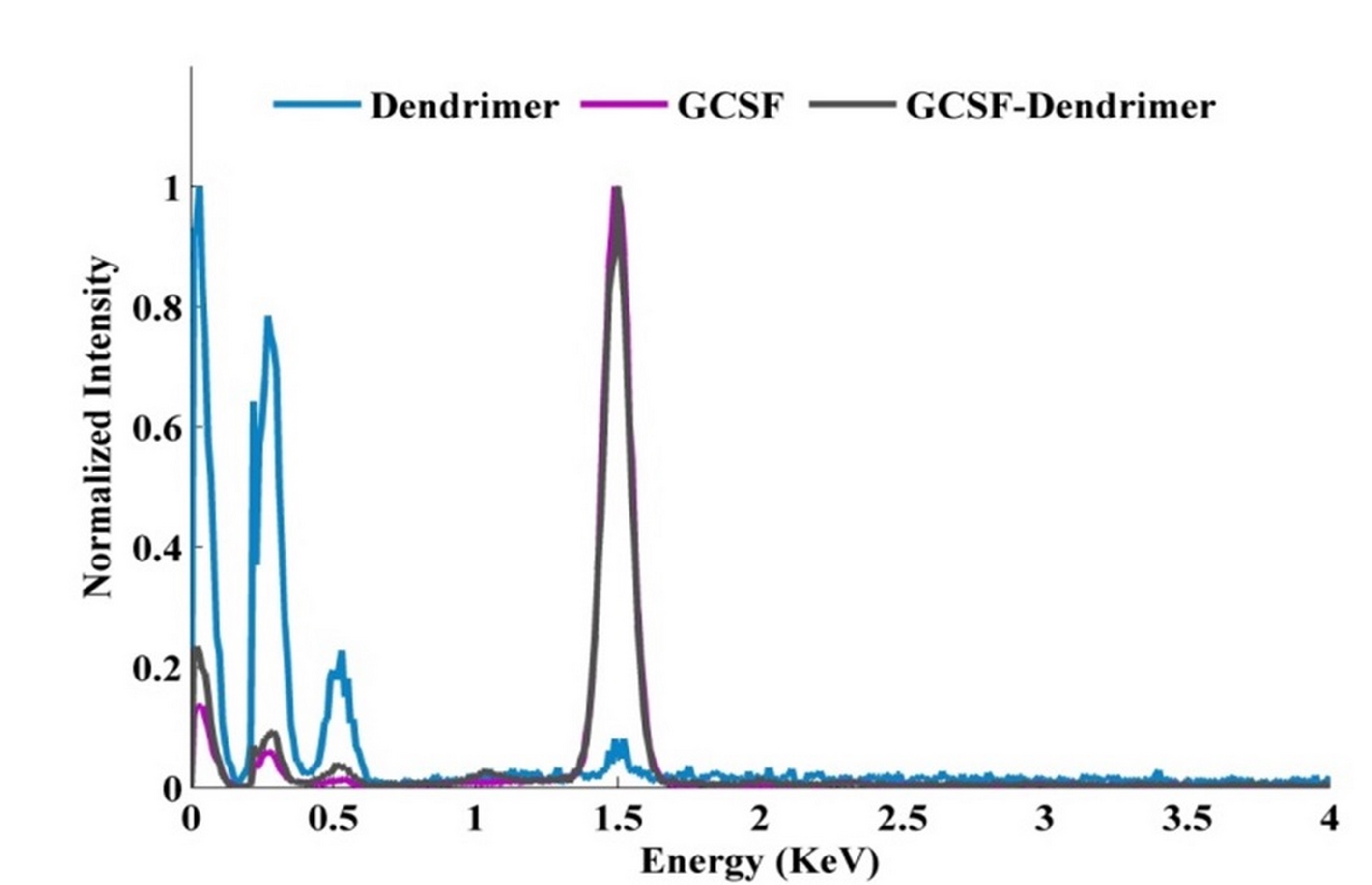

Supplement: Supplementary file 3 — Figure S3 [file PRP2-9-e00826-s009.jpg]

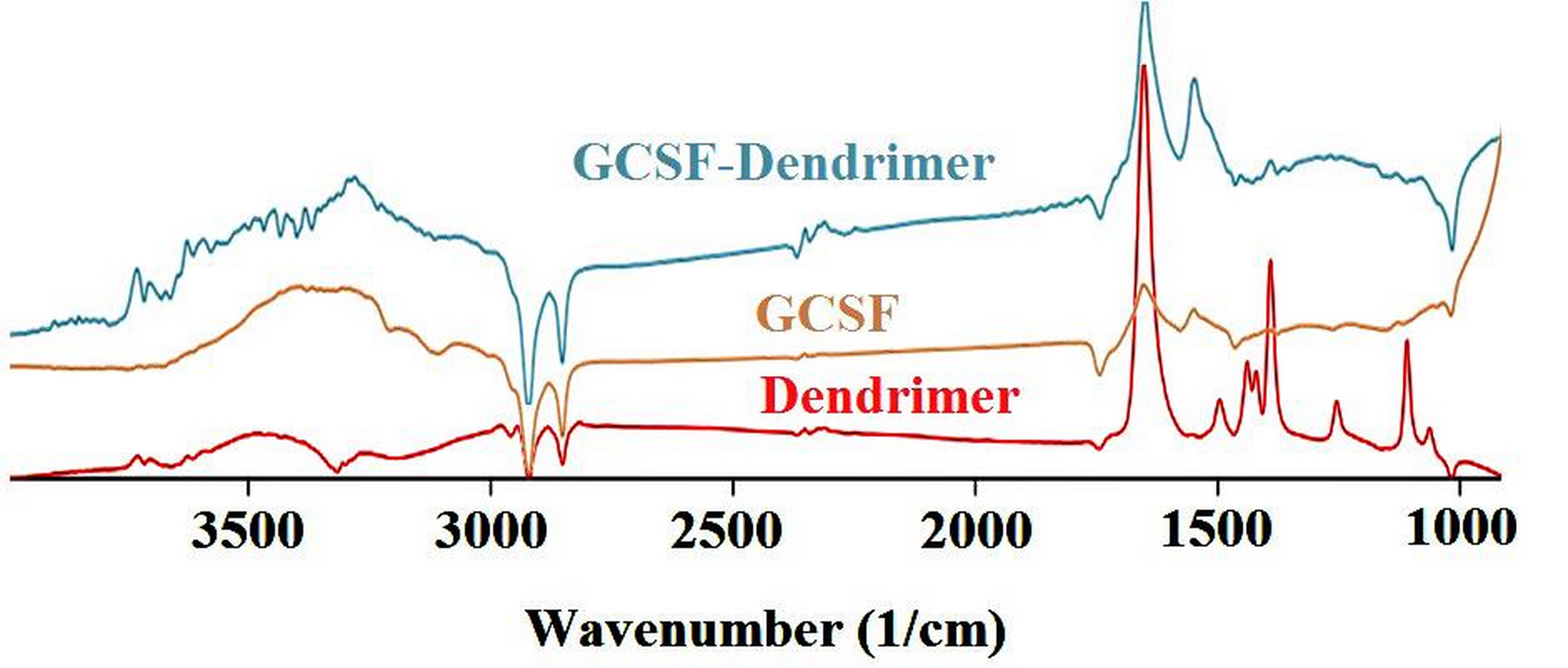

Supplement: Supplementary file 4 — Figure S4 [file PRP2-9-e00826-s004.jpg]

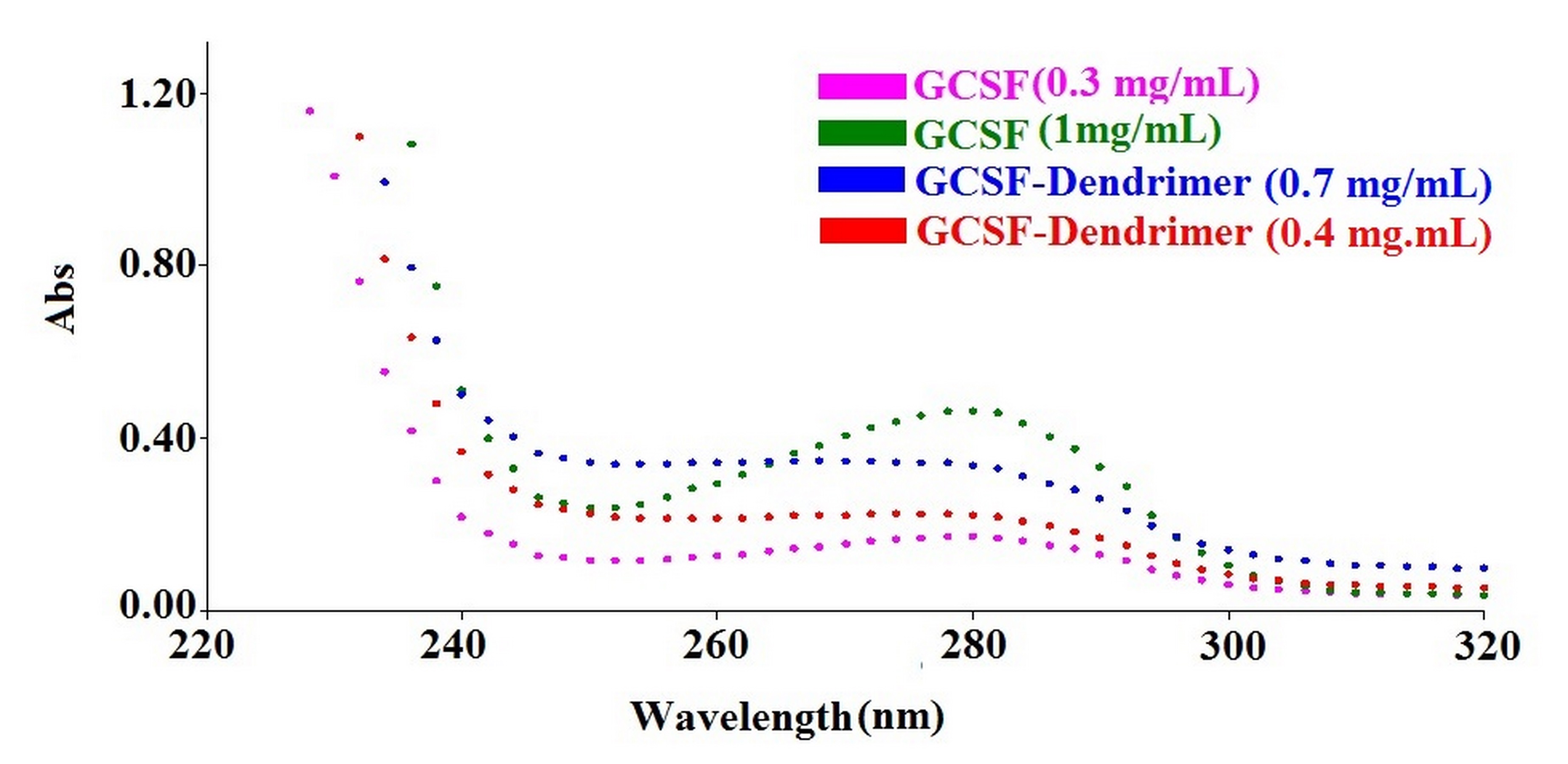

Supplement: Supplementary file 5 — Figure S5 [file PRP2-9-e00826-s011.jpg]

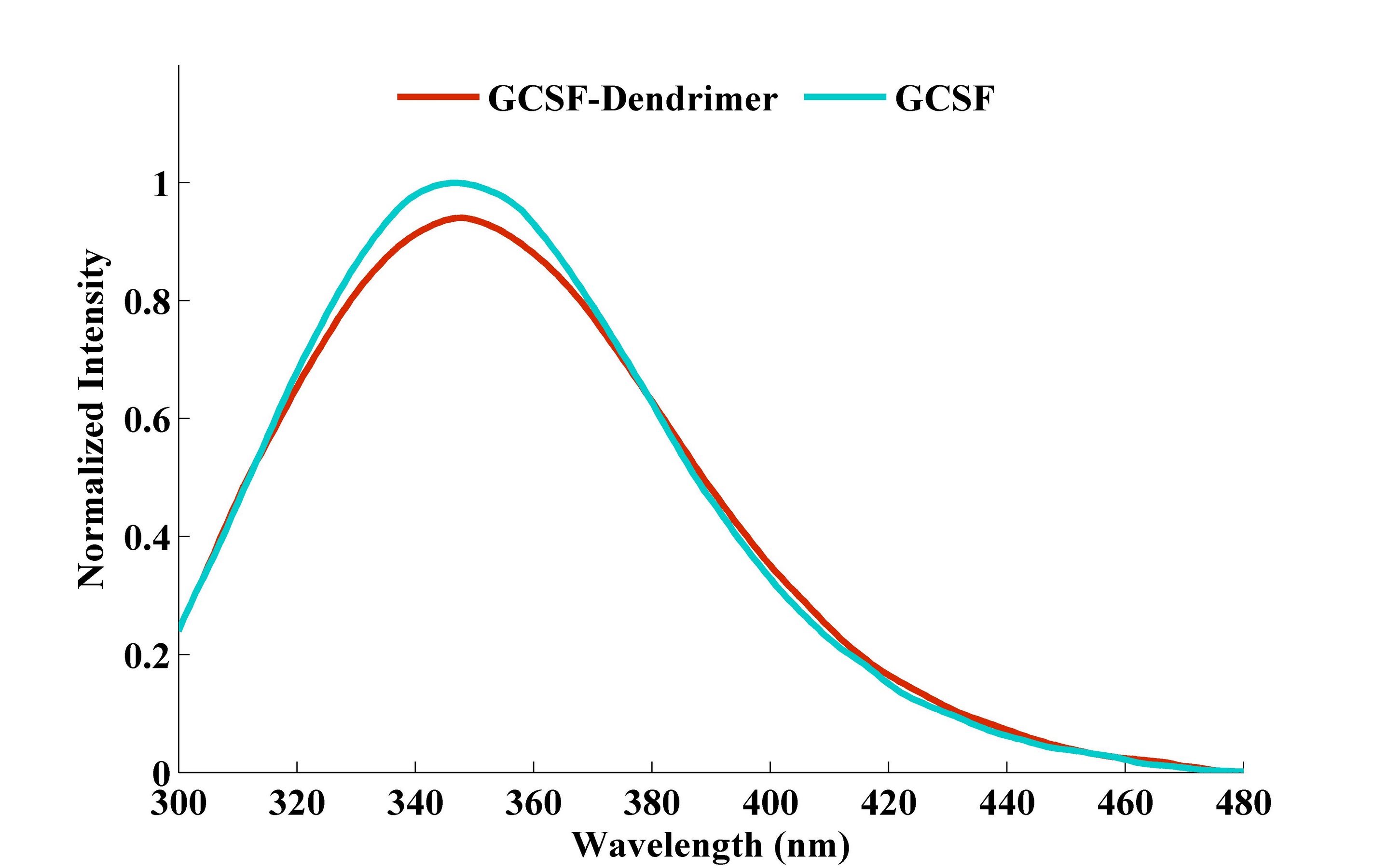

Supplement: Supplementary file 6 — Figure S6 [file PRP2-9-e00826-s002.jpg]

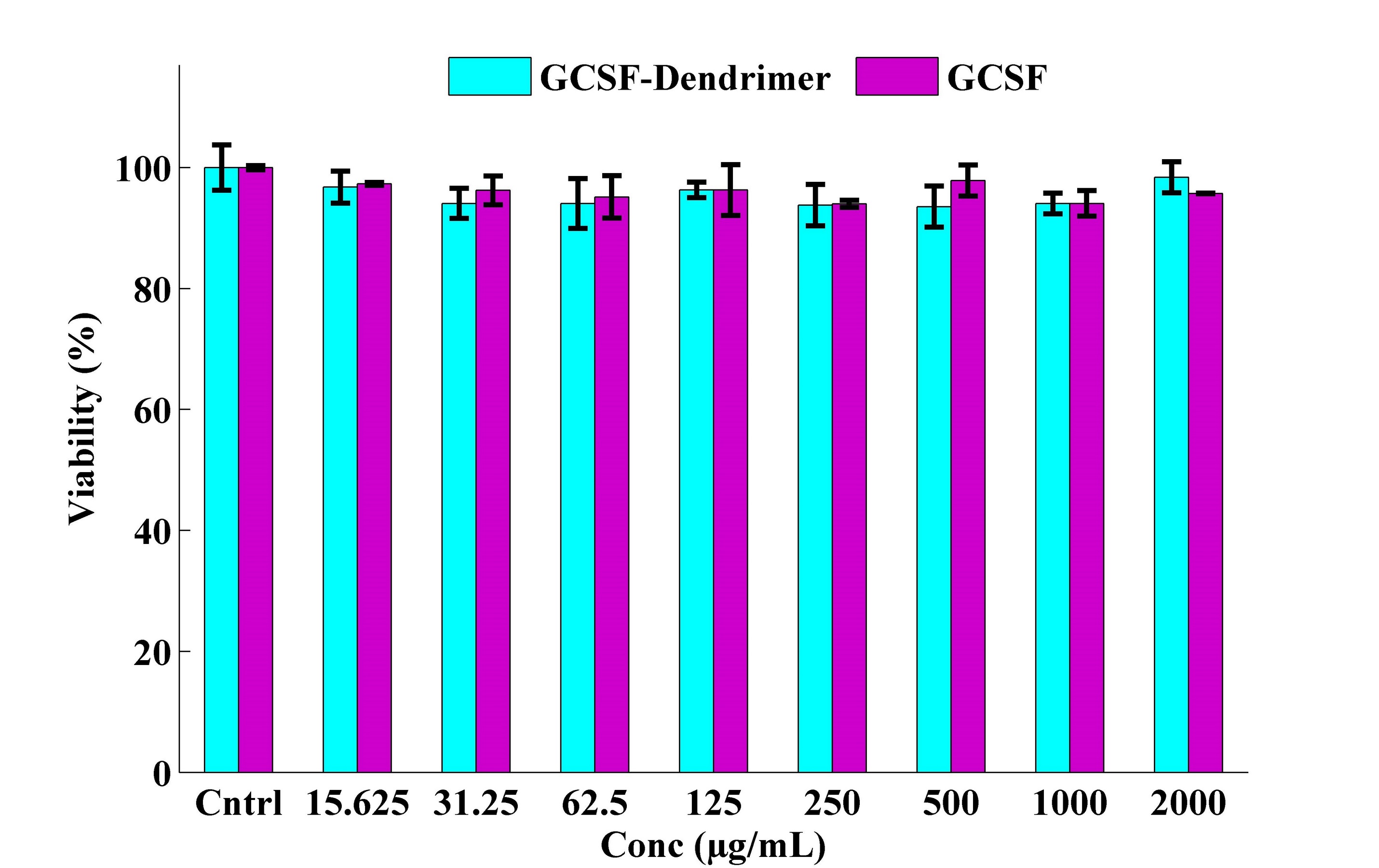

Supplement: Supplementary file 7 — Figure S7 [file PRP2-9-e00826-s006.jpg]

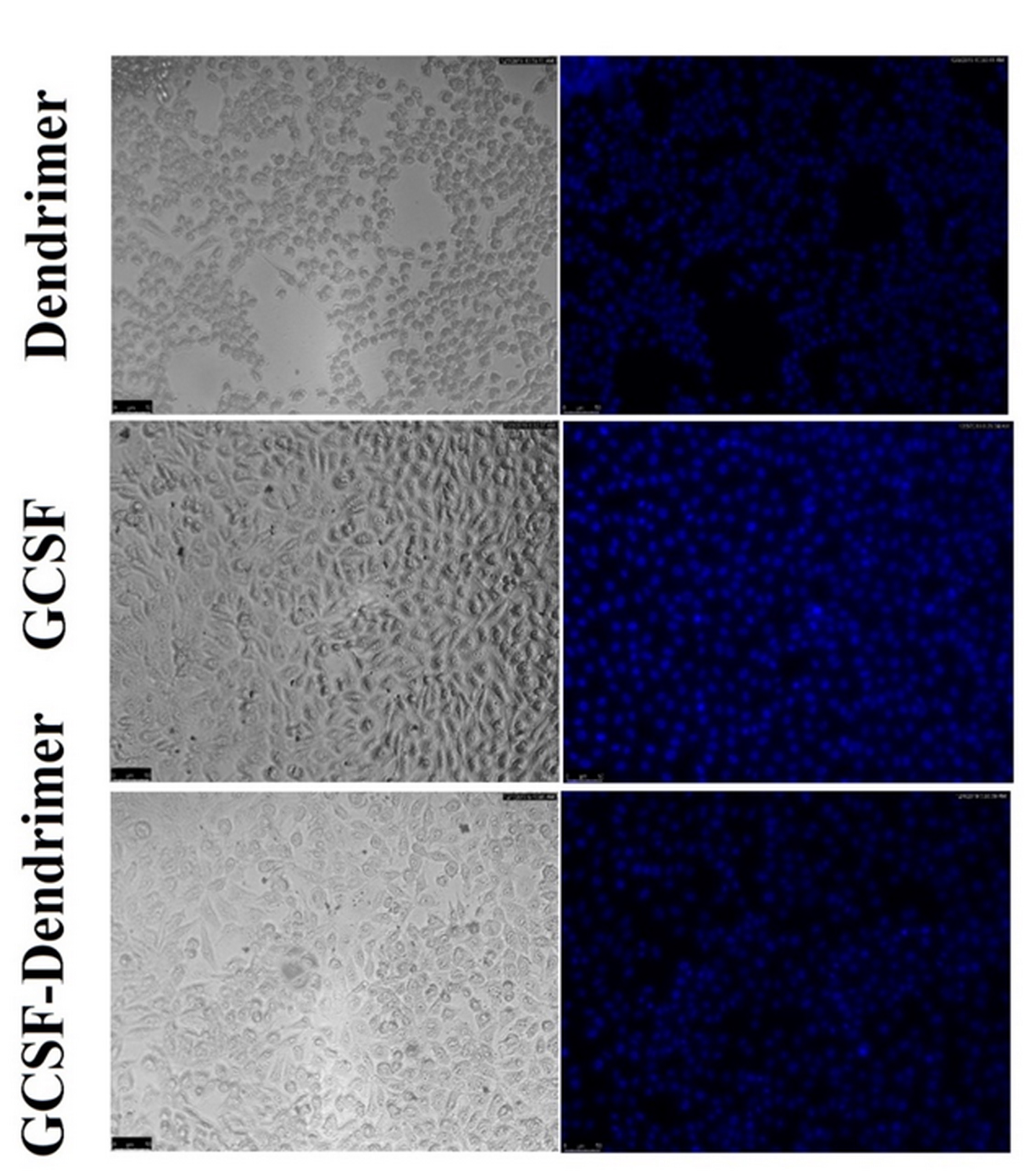

Supplement: Supplementary file 8 — Figure S8 [file PRP2-9-e00826-s007.jpg]

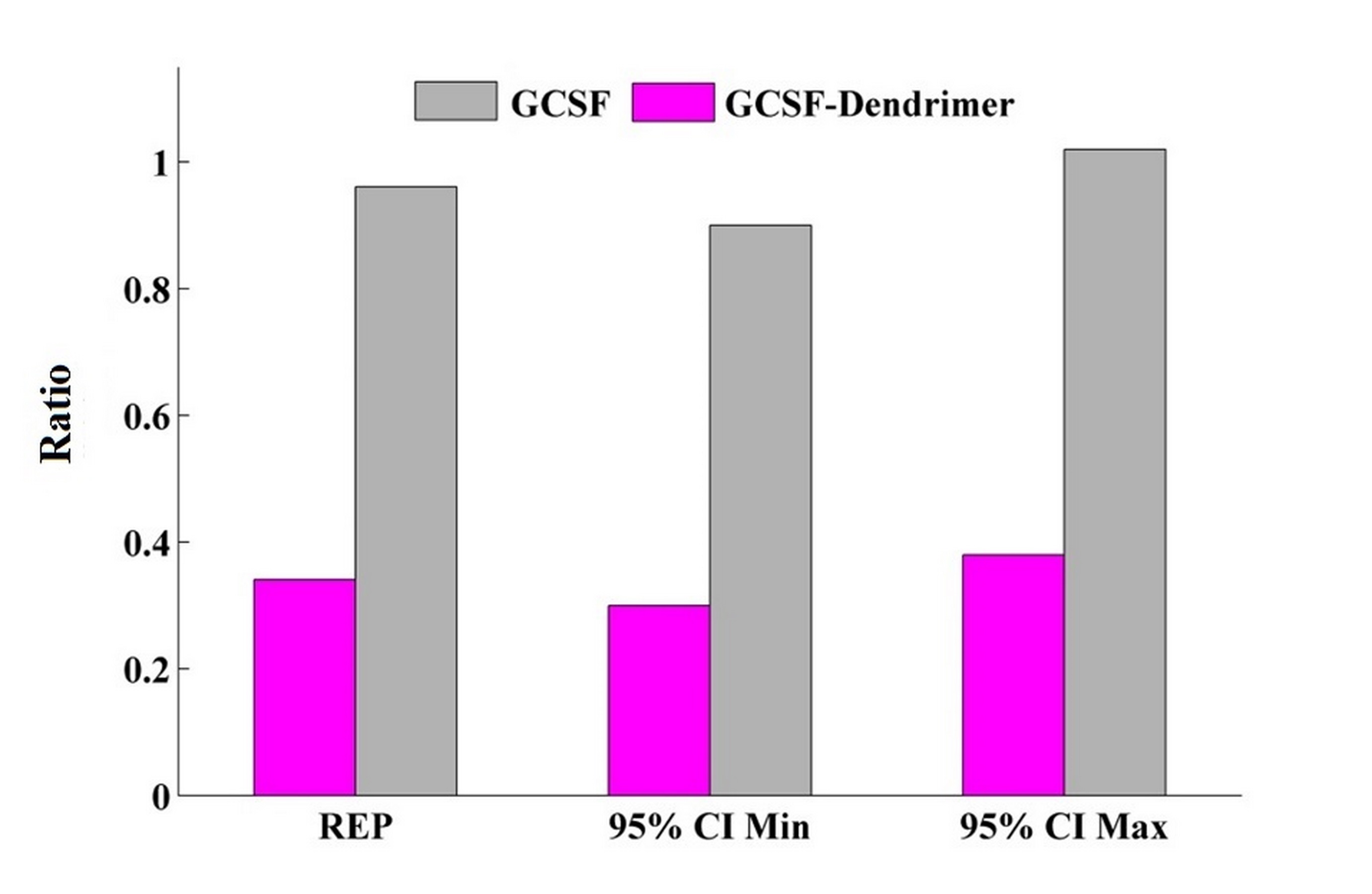

Supplement: Supplementary file 9 — Figure S9 [file PRP2-9-e00826-s001.jpg]

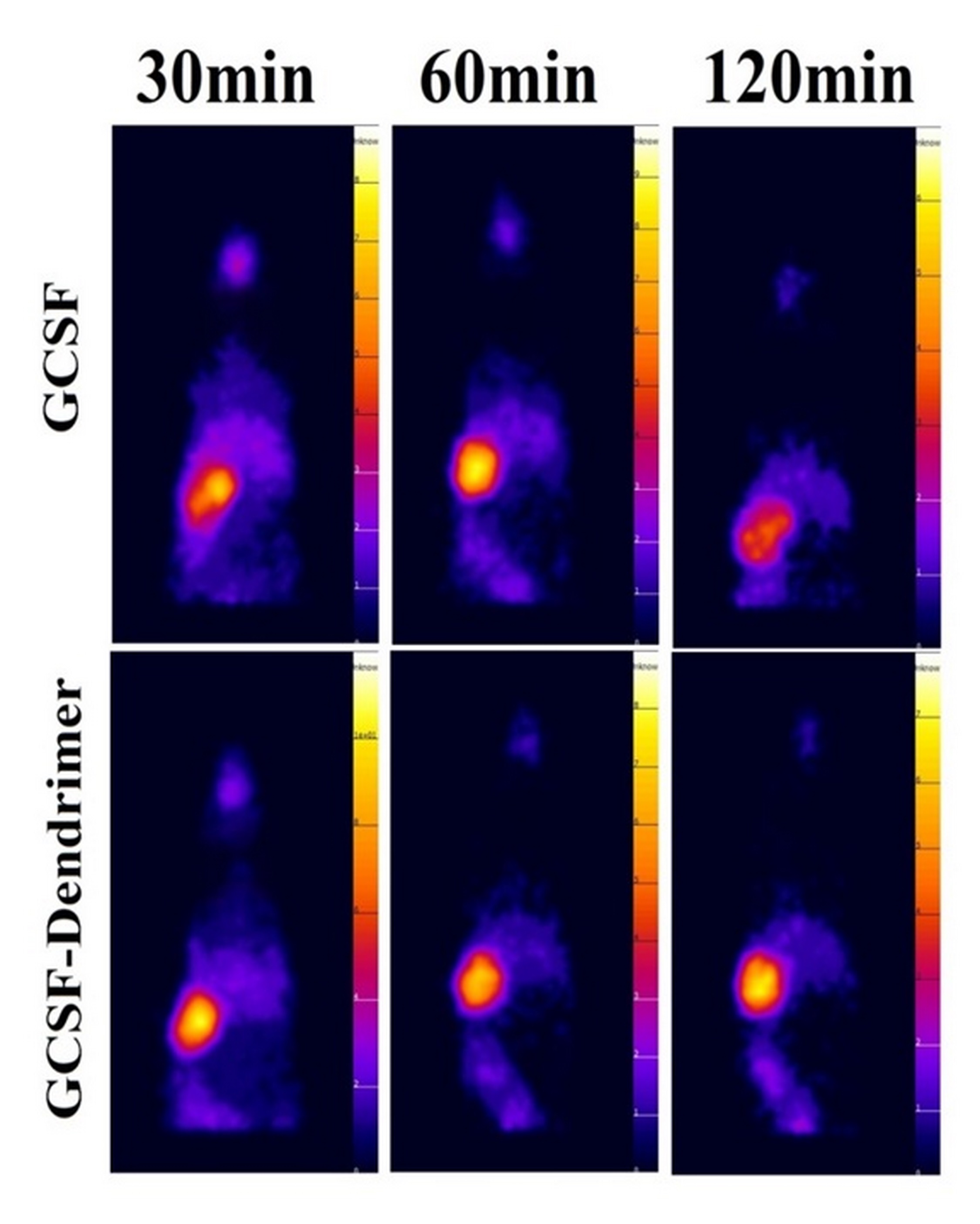

Supplement: Supplementary file 10 — Figure S10 [file PRP2-9-e00826-s005.jpg]
