# Supplementary material for: Novel G‐CSF conjugated anionic globular dendrimer: Preparation and biological activity assessment
Source: Pharmacol Res Perspect. 2021 Jul 16;9(4):e00826. doi: 10.1002/prp2.826 (PMC8283867; doi:10.1002/prp2.826)
Supplement: Supplementary file 11 — Supplementary Material [file PRP2-9-e00826-s003.docx]

**Novel G-CSF Conjugated Anionic Globular Dendrimer: Preparation and Biological Activity Assessment**

Seyed Shahaboddin Mousavi Motlagh^a^, Mohammad Seyedhamzeh^b^, Reza Ahangari Cohan^a*^, Mehdi Shafiee Ardestani^b*^, Behrouz Vaziri ^c^, Kayhan Azadmanesh ^d^, Sahar Saberi^e^, Vahideh Masomi^e^

^a^ Department of Nanobiotechnology, New Technologies Research Group, Pasteur Institute of Iran, Tehran, Iran

^b^ Faculty of Pharmacy, Tehran University of Medical Sciences, Tehran, Iran

^c^ Biotechnology Research Center, Pasteur Institute of Iran, Tehran, Iran

^d^ Department of Molecular Virology, Pasteur Institute of Iran, Tehran, Iran

^e^ Department of Biotechnology, Food and Drug Control Laboratories, National Food and Drug Organization, Tehran, Iran

**
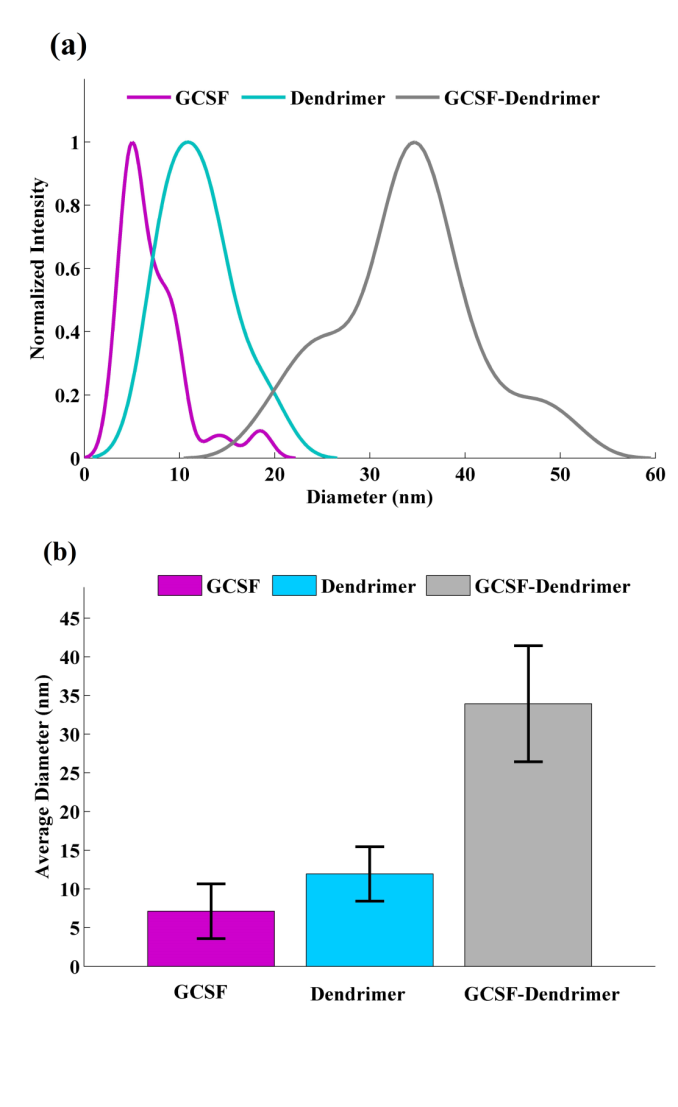
**

**Figure S1.** (a) Size distribution and (b) average size obtained from AFM images. Data are represented as mean ± standard deviation from three replicates.

**
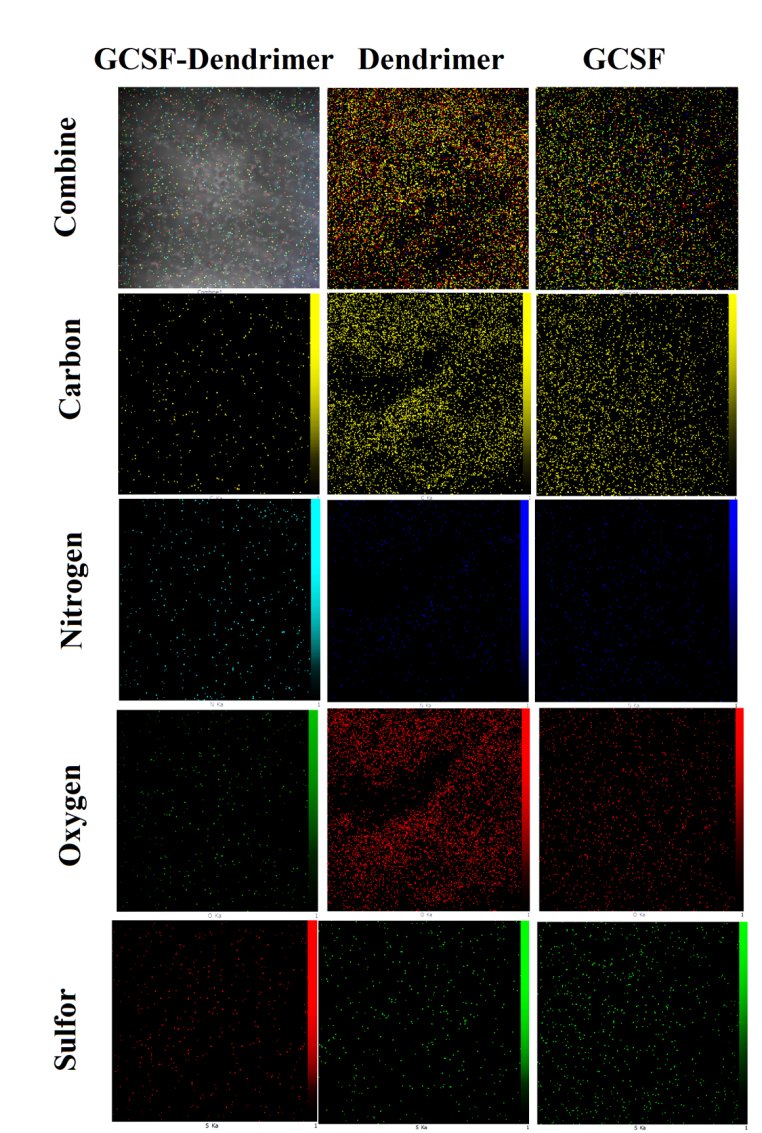
**

**Figure S2.** Elemental map results of the dendrimer, G-CSF, and G-CSF-dendrimer.

**
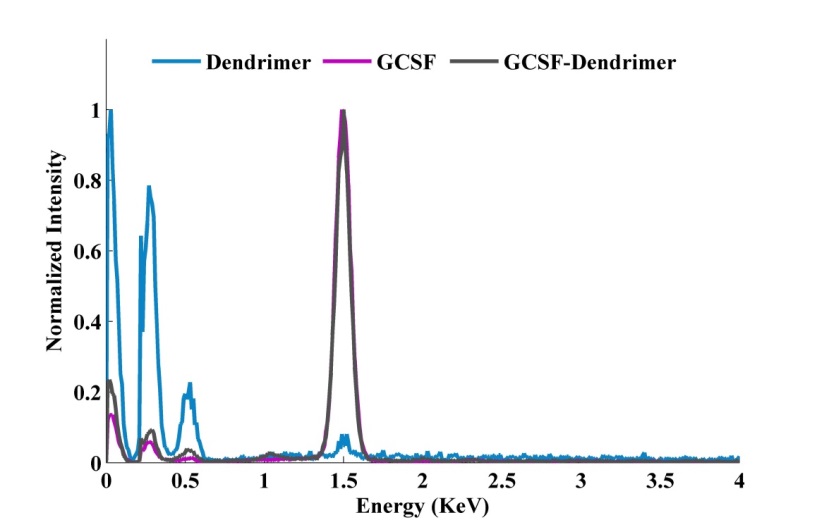
**

**Figure S3.** EDS results of the dendrimer (light blue), G-CSF (purple), and G-CSF-dendrimer (gray).


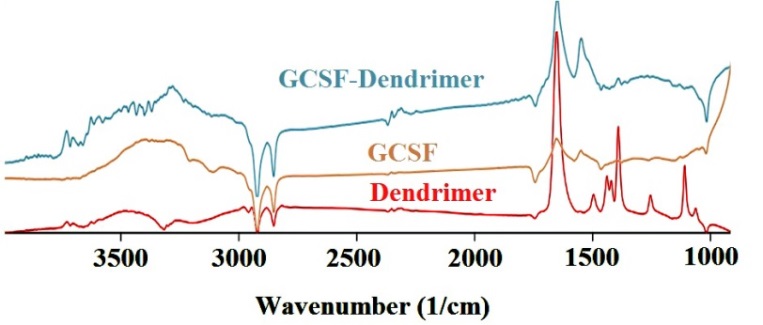


**Figure S4.** Biologic FTIR spectrum of the dendrimer (red), G-CSF (brown), and G-CSF-dendrimer (blue)**.**

**
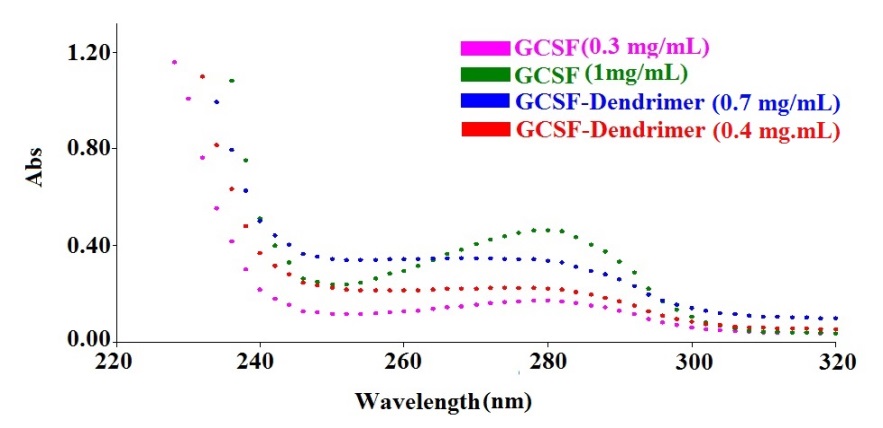
**

**Figure S5.** UV/Vis spectrum of G-CSF and G-CSF-dendrimer at different concentrations.

**
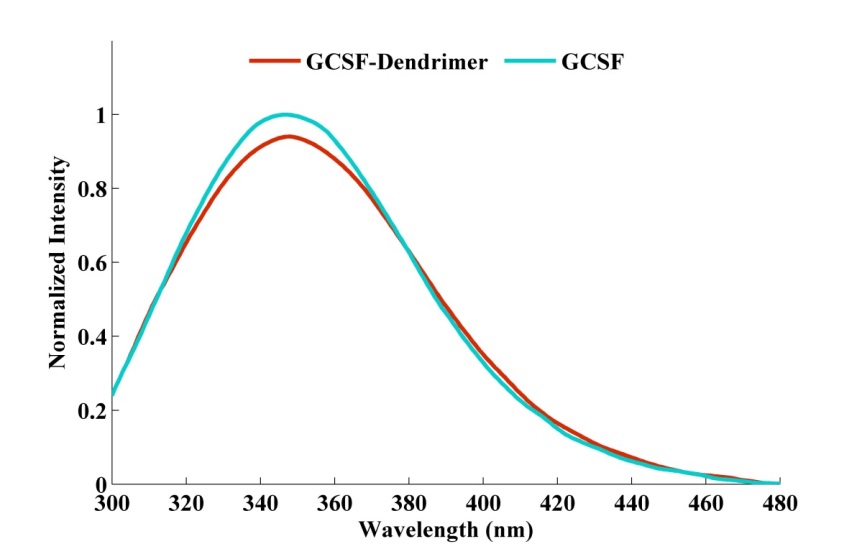
**

**Figure S6.** Fluorescence spectrum of G-CSF (blue) and G-CSF-Dendrimer (red).

**
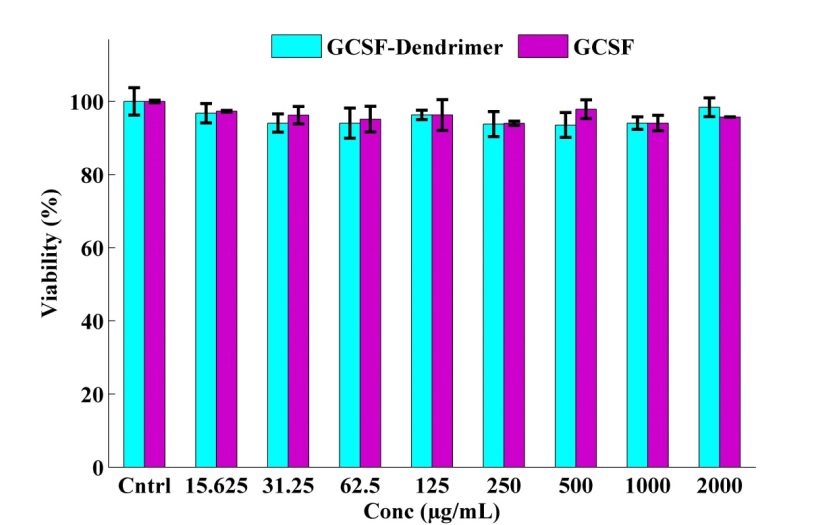
**

**Figure S7.** XTT assay results of G-CSF (purple) and G-CSF-Dendrimer (blue) on L929 cell lines after 48 h. Data are represented as mean ± standard deviation from four independent replicates.

**
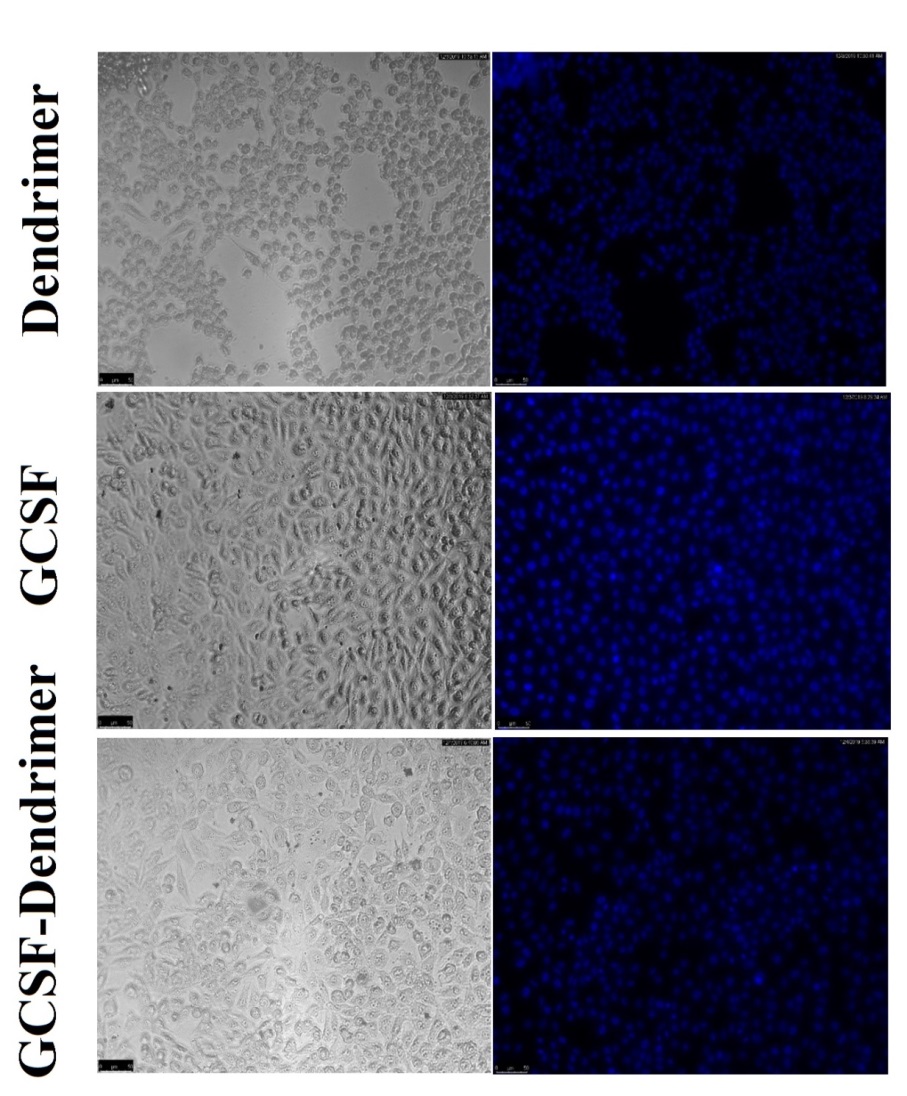
**

**Figure S8.** Inverted microscope (left) and fluorescence (right) images of Hoechst stained L929 cells after treatment with the dendrimer, G-CSF, and G-CSF-Dendrimer (100X).

**
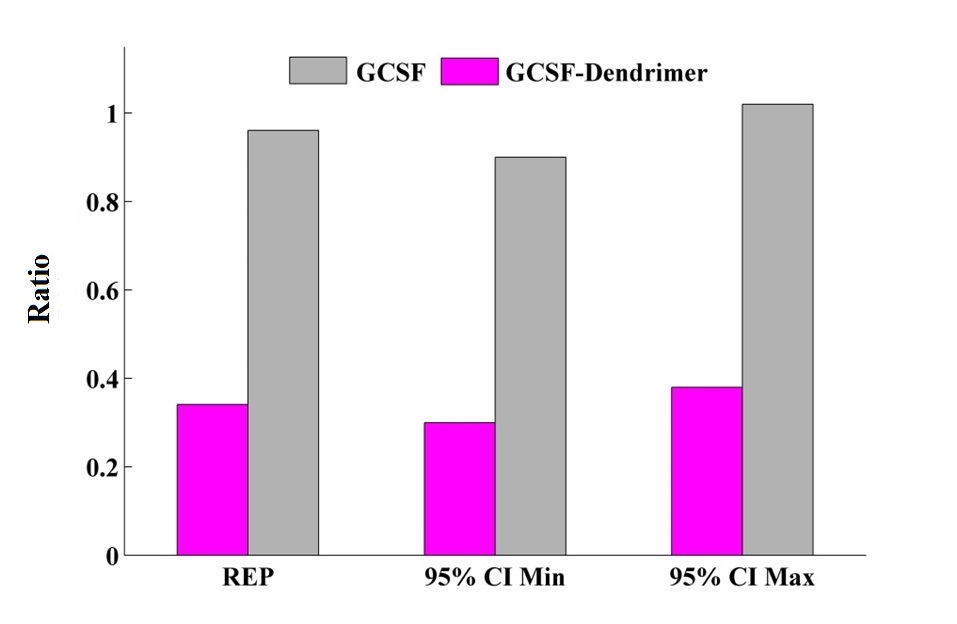
**

**Figure S9.** Effects of G-CSF and G-CSF-dendrimer on NFS60 cell lines after 48 h treatment (relative estimated potency against W.H.O standard (NIBSC) with a confidence interval of 95%). Data are represented as mean from four independent replicates.

**
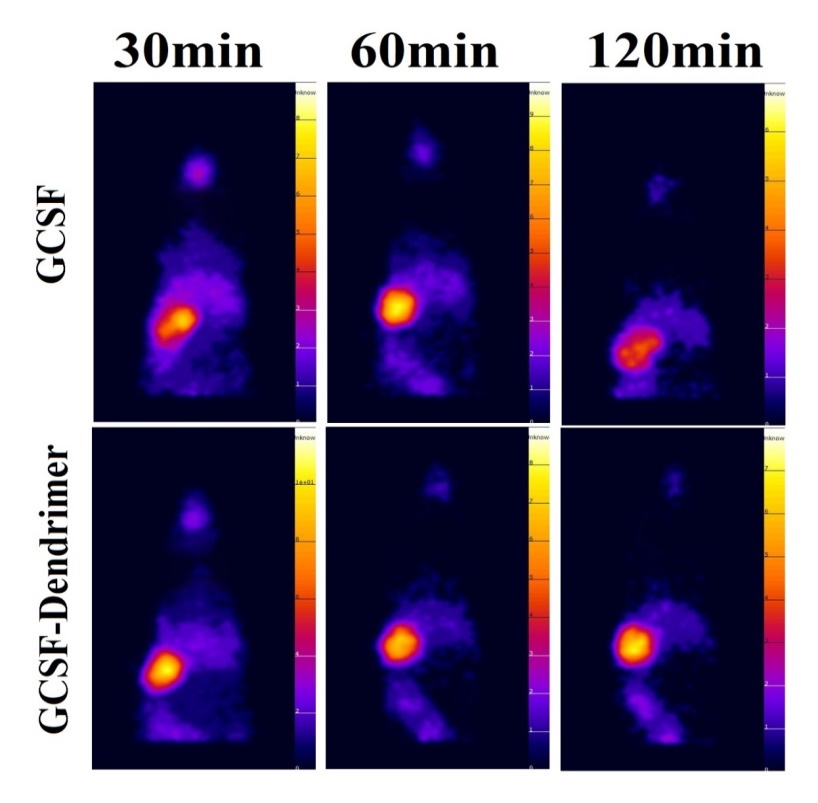
**

**Figure S10.** SPECT images of rats obtained from injection of G-CSF and G-CSF-Dendrimer at different times (30 min, 60 min, and 120 min).
